# Microbial collagenase activity is linked to oral–gut translocation in advanced chronic liver disease

**DOI:** 10.1038/s41564-025-02223-0

**Published:** 2025-12-29

**Authors:** Shen Jin, Aurelie Cenier, Daniela Wetzel, Bethlehem Arefaine, Mar Moreno-Gonzalez, Marilena Stamouli, Merianne Mohamad, Mariia Lupatsii, Emilio Ríos, Sunjae Lee, Ane Zamalloa, Shilpa Chokshi, Adil Mardinoglu, Saeed Shoaie, Naiara Beraza, Vishal C. Patel, Melanie Schirmer

**Affiliations:** 1 Translational Microbiome Data Integration, School of Life Sciences, Technical University of Munich, Freising, Germany; 2 Roger Williams Institute of Liver Studies, School of Immunology & Microbial Sciences, Faculty of Life Sciences and Medicine, King’s College London, Foundation for Liver Research and King’s College Hospital, London, UK; 3 Food Microbes and Health Institute Strategic Programme, Quadram Institute Bioscience, Norwich, UK; 4 Centre for Host–Microbiome Interactions, Faculty of Dentistry, Oral & Craniofacial Sciences, King’s College London, London, UK; 5 School of Life Sciences, Gwangju Institute of Science and Technology, Gwangju, Korea; 6 Institute of Liver Studies, King’s College Hospital NHS Foundation Trust, London, UK; 7 Centre of Environmental Hepatology, Peninsula Medical School Faculty of Health, University of Plymouth, Plymouth, UK; 8 Science for Life Laboratory, KTH—Royal Institute of Technology, Stockholm, Sweden; 9 Center for Organoid Systems, Technical University Munich, Garching, Germany; 10 ZIEL—Institute for Food & Health, Technical University of Munich, Freising, Germany

**Keywords:** Microbiome, Metagenomics

## Abstract

Microbiome perturbations are associated with advanced chronic liver disease (ACLD), but how microorganisms contribute to disease mechanisms is unclear. Here we analysed metagenomes of paired saliva and faecal samples from an ACLD cohort of 86 individuals, plus 2 control groups of 52 healthy individuals and 14 patients with sepsis. We identified highly similar oral and gut bacterial strains, including *Veillonella* and *Streptococcus* spp., which increased in absolute abundance in the gut of patients with ACLD compared with controls. These microbial translocators uniquely share a *prtC* gene encoding a collagenase-like proteinase, and its faecal abundance was a robust ACLD biomarker (area under precision-recall curve = 0.91). A mouse model of hepatic fibrosis inoculated with *Veillonella* and *Streptococcus prtC-*encoding patient isolates showed exacerbation of gut barrier impairment and hepatic fibrosis. Furthermore, faecal collagenase activity was increased in patients with ACLD and experimentally confirmed for the *prtC* gene of translocating *Veillonella parvula*. These findings establish mechanistic links between oral–gut translocation and ACLD pathobiology.

## Abstract (web summary)

While oral bacteria rarely colonize the healthy gut, they dominate the gut microbiome of patients with chronic liver disease. Here a bacterial gene encoded by microbial translocators is linked to gut barrier disruption and fibrosis.

## Main

Advanced chronic liver disease (ACLD), a global disease burden with over two million deaths every year^1^, results from the histological development of cirrhosis^2^. During chronic liver inflammation, normal hepatic parenchyma is replaced by scar tissue and regenerative nodules^3,4^. The first stage is compensated ACLD (cACLD), in which hepatic synthetic function is maintained and patients are mostly asymptomatic. Subsequently, acutely decompensated ACLD (AD) is driven by worsening portal hypertension and portosystemic shunting and characterized by synthetic failure^5^. The most severe form is acute-on-chronic liver failure (ACLF), which occurs in patients with cACLD and AD and is marked by systemic inflammation, (multi-)organ failure and high short-term mortality (30-day mortality rate in >57% of patients)^6,7^. Current standard of care targets complications, while interventions to prevent disease progression^8^ and new approaches for early diagnosis are lacking^9,10^.

Gut microbial perturbations are associated with ACLD pathobiology, in which diversity is decreased and characterized by a loss of bacterial commensals, such as Lachnospiraceae, Bacteroidaceae and Ruminococcaceae, while opportunistic pathogens increase in abundance, including Enterobacteriaceae, Enterococcaceae, Veillonellaceae and Streptococcaceae^11–18^. Microbial factors can also predict ACLD^19^, in which the abundances of 19 species^20^ can distinguish between patients with cirrhosis and healthy controls (area under the receiver operating characteristic curve (auROC) = 0.86). Furthermore, antibiotic resistance genes^21^ and microbially produced aromatic and branched-chain amino acids^20^ are associated with ACLD, while increased microbial fatty acid biosynthesis and glycan degradation activity are associated with cirrhosis severity and hepatic damage^22^. This implicates microbiome perturbations in disease; however, mechanistic insights into how this contributes to ACLD pathobiology are missing.

Recently, increases in bacteria typical of oral cavities were identified, in which *Veillonella*, *Streptococcus* and *Prevotella* spp. are found in high relative abundance in the faeces of patients with ACLD^12,23^. This was also reported for other diseases, including ulcerative colitis^24^, colorectal cancer^25,26^, rheumatoid arthritis, type 1 diabetes^27,28^ and hypertension^29^, suggesting that microbiome oral–gut translocation may have an important role in these diseases^30,31^. Rifaximin-α, a gut-targeted therapy to prevent hepatic encephalopathy (HE) in AD, reduces oralization of the gut microbiome and suppresses bacteria with intestinal mucus degradation capabilities, potentially ameliorating HE symptoms by promoting gut barrier integrity^32^. Another bacterium typical of the oral cavity, *Veillonella parvula*, degrades immunosuppressive thiopurine drugs potentially impacting therapeutic efficacy^24^. However, it is unclear whether these bacteria actually translocate from the mouth and colonize the gut during disease or whether this increase is due to decreased total gut bacterial load^33^. Paired saliva and faecal samples with metagenomic strain profiling are essential to investigate the oral origin of atypical gut bacteria, but these data are currently lacking for most cohort studies.

Here we investigated the role of oral–gut translocation in ACLD using metagenomic data from paired saliva and faecal samples. Using reference- and assembly-based analyses, we computationally identified co-occurring bacterial strains in saliva and faeces, obtained viable next-to-identical clinical isolates and experimentally confirmed increases in absolute abundance of oral microorganisms in ACLD faecal samples. By integrating microbiome strain and functional profiles with detailed clinical data, including disease severity indices (Child–Pugh and Model for End-Stage Liver Disease (MELD) scores) and a small-intestinal epithelial cell damage marker (plasma fatty acid binding protein 2 (FABP2)), we identified a bacterial collagenase-like proteinase (*prtC* encoded) associated with disease pathobiology. This *prtC* gene was uniquely shared by oral–gut translocating bacteria, and its faecal abundance emerged as a robust ACLD biomarker (area under precision-recall curve (auPR) = 0.91, auROC = 0.89; external validation cohort: auPR = 0.93, auROC = 0.93). With the use of a preclinical mouse model of hepatic fibrosis, carbon tetrachloride (CCl_4_)-treated mice were gavaged with oral patient isolates of *Veillonella* and *Streptococcus* spp. resulting in exacerbated gut barrier impairment (including increases in colonic albumin levels and mislocalization of E-cadherin and occludin), increased hepatic and intestinal fibrosis and small-intestinal bacterial overgrowth (SIBO). Collagenase activity was also increased in the faecal supernatant of patients with ACLD compared with that of healthy controls. Furthermore, in vitro expression of the *V. parvula prtC* gene in *Escherichia coli* confirmed collagenase activity of the implicated strains. Collectively, our study links microbial strains and genes involved in oral–gut translocation to ACLD pathobiology.

## Results

### The oral microbiome shifts at early disease stages and oral–gut similarity increases with ACLD progression

**Fig. 1:**
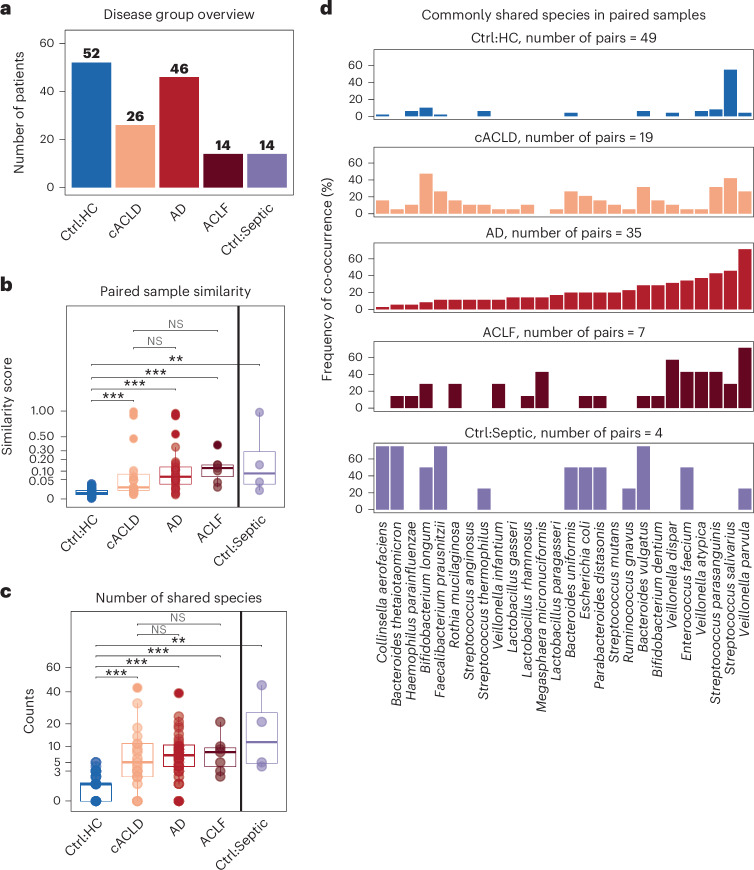
Oral–gut translocation increases as ACLD severity worsens. **a**, Cohort overview. Number of patients per disease group. Patients with ACLD were stratified according to disease stage ranging from cACLD and AD to ACLF. Two control groups were included: healthy patients (Ctrl:HC) and patients with sepsis (Ctrl:Septic). **b**, Similarity (1 − Bray–Curtis dissimilarity) between paired faecal and saliva samples stratified by disease group (two-sided Wilcoxon, NS = not significant, *P* value adjusted with BH). **c**, Number of shared species in paired faecal and saliva samples increased significantly as disease severity increased (two-sided Wilcoxon, based on reference-based species profiles, *P* value adjusted with BH). Adjusted *P* values: ***P < 0.001; **P = (0.001, 0.01]; *P = (0.01–0.05]; NS, *P* > 0.05. **d**, Identification of microbial species commonly shared between saliva and faecal samples. The *y*-axis indicates the percentage of samples in which the species was detected in both paired sample types. Each column represents a species ordered by their frequency in the AD group. Source data

Saliva and faecal samples from 86 patients with ACLD and 2 control groups consisting of 52 healthy participants and 14 patients with sepsis with no underlying cirrhosis were analysed (Fig. 1a and Table 1 for clinical characteristics). Patients with sepsis formed our second control group as they showed similar characteristics in hospitalization, age, antibiotic usage and proton-pump inhibitor exposure compared with patients with ACLD. Patients with ACLD were grouped based on disease severity, including patients with cACLD, AD and ACLF, and metagenomic sequencing data from 266 samples (*n*_faecal_ = 143, *n*_saliva_ = 123) were used to identify taxonomic and functional microbiome signals associated with oral–gut translocation.

**Table 1:** Summary of clinical characteristics of study groups

Parameters		Ctrl:HC (*n* = 52)	SC (*n* = 26)	AD (*n* = 46)	ACLF (*n* = 14)	Ctrl:Septic (*n* = 14)	*P* value
**General**	Age (years)	34 (22–70)	61 (50–67)	55 (46–64)	47 (41–54)	61 (50–65)	<0.0001
	Male gender (number (%))	21 (42.0%)	18 (69.2%)	33 (71.7%)	9 (64.3%)	11 (78.6%)	0.3739
**Aetiology of cirrhosis**	Alcohol-related liver disease (*n* (%))	N/A	13 (50%)	29 (63.0%)	10 (71.4%)	N/A	0.3637
	NAFLD and NASH (*n* (%))	N/A	2 (7.7%)	8 (17.4%)	1 (7.1%)	N/A	0.3908
	Cholestatic and autoimmune (*n* (%)) PBC, PSC, secondary biliary cirrhosis and AIH	N/A	5 (19.2%)	4 (8.7%)	1 (7.1%)	N/A	0.3462
	Metabolic (*n* (%)) Wilson’s disease, haemochromatosis and A1AT deficiency	N/A	0	1 (2.2%)	0	N/A	0.8798
	Other (*n* (%)) ‘Cryptogenic’, treated viral hepatitis and chronic veno-occlusive-related cirrhosis (Budd–Chiari)	N/A	6 (23.1%)	4 (8.7%)	2 (14.3%)	N/A	0.2390
**Clinical features at enrolment**	Presence of ascites (*n* (%))	N/A	3 (11.5%)	35 (76.1%)	10 (71.4%)	N/A	<0.0001
	Presence of hepatic encephalopathy (*n* (%))	N/A	0	4 (8.7%)	6 (42.9%)	0	0.0002
**Antibacterial therapy at enrolment** **(*n*** **(%))**	Antibiotics—any	N/A	7 (26.9%)	33 (71.7%)	14 (100%)	14 (100%)	<0.0001
	Antifungal—any	N/A	0	2 (4.4%)	3 (21.4%)	4 (28.6%)	0.0047
**Other pharmacotherapy at enrolment** **(*n*** **(%))**	Proton pump inhibitors (omeprazole, lansoprazole)	N/A	11 (42.3%)	26 (56.5%)	9 (64.3%)	5 (35.7%)	0.3024
	Lactulose	N/A	5 (19.2%)	17 (37.0%)	9 (64.3%)	2 (14.3%)	0.0119
	H2 antagonists (ranitidine)	N/A	0	7 (15.2%)	1 (7.1%)	0	0.0804
**Disease severity and prognostic scores** **(*n*)**	Child–Pugh	N/A	5 (5–6)	9 (7–10)	11 (7–13)	N/A	<0.0001
	MELD	N/A	8 (8–11)	19 (14–25)	31 (26–39)	N/A	<0.0001
	CLIF-C-AD^a^	N/A	43 ± 7	49 ± 7	61 ± 10	N/A	<0.0001
	CLIF-C-AD ACLF	N/A	N/A	N/A	52 ± 13	N/A	–
	SOFA	N/A	3 (3–5)	7 (5–8)	12 (8–15)	7 (6–9)	<0.0001
**Mortality**	30-day (*n* (%))	N/A	0	1 (2.2%)	2 (14.3%)	2 (14.3%)	0.0651
**Transplantation**	30-day (*n* (%))	N/A	0	4 (8.7%)	1 (7.1%)	N/A	0.3092

*P* values were calculated using one-way ANOVA.

PBC, primary biliary cholangitis; PSC, primary sclerosing cholangitis; AIH, autoimmune hepatitis; A1AT, alpha-1-antitrypsin; NAFLD, non-alcoholic fatty liver disease; NASH, non-alcoholic steatohepatitis; N/A, not applicable; MELD, model for end-stage liver disease; UKELD, United Kingdom model for end-stage liver disease; CLIF-C-AD, CLIF Consortium acute decompensation score; CLIF-C-ACLF, CLIF Consortium acute-on-chronic liver failure score; CLIF-C OF, CLIF Consortium organ failure score; SOFA, sequential organ failure assessment score; CLIF, Chronic Liver Failure Consortium; ACLF, acute-on-chronic liver failure.

^a^Normally distributed values presented as mean ± s.d.; non-normally distributed values are presented as median (interquartile range).

**Extended Data Fig. 1:**
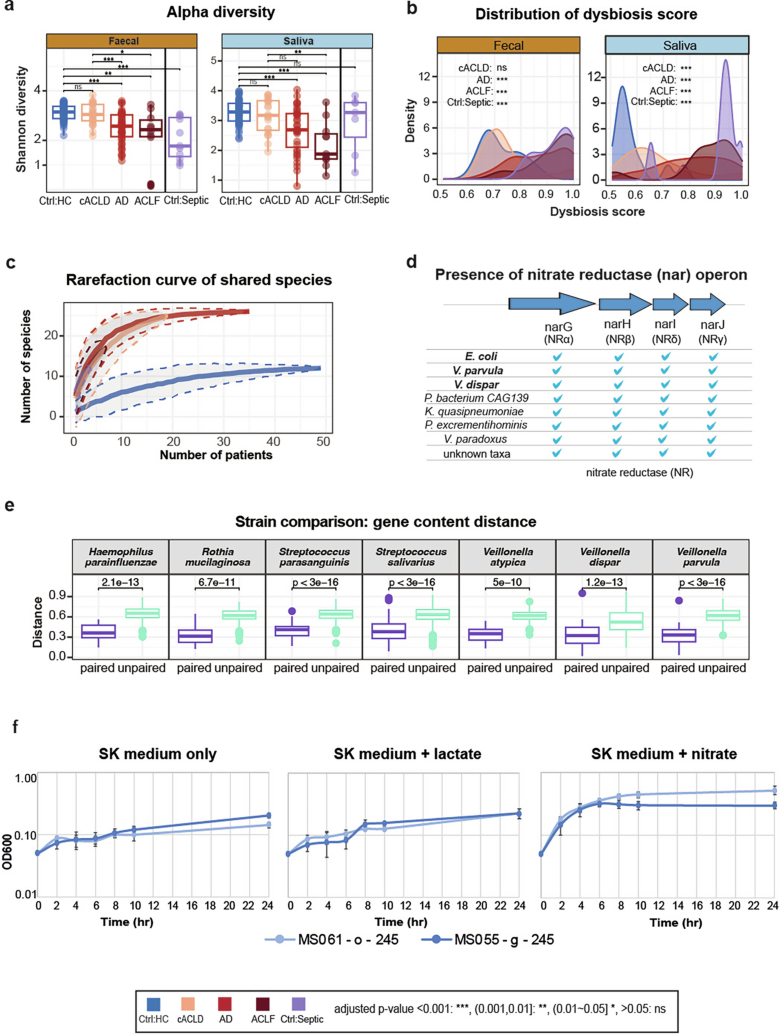
Identification of oral-gut translocators from paired faecal and saliva samples. (**a**) Alpha diversity (Shannon index) decreased in faeces and saliva as ACLD severity increased (two-sided Wilcoxon, ns = not significant, p-value adjusted with Benjamini Hochberg [BH]). (**b**) Dysbiosis score distribution of faecal (left) and saliva (right) microbiome (two-sided Wilcoxon). (**c**) Rarefaction curves showing number of shared species against number of patients for each disease group (95% confidence interval). (**d**) Presence of the nitrate reductase (nar) operon in metagenomic assembled pangenomes (MSPs). The presence of four key genes of the nar operon were examined. Species with all narGHJI genes ( > 30% identity >80% coverage) are shown and species commonly shared between faecal and saliva samples are marked in bold. (**e**) Comparison of gene content similarity between strains from paired versus unpaired faecal and saliva samples. Y-axis reflects binary distance estimates based on gene presence/absence in the respective pangenome (PanPhlAn). (**f**) Next-to-identical *V. parvula* strains from paired faecal and saliva samples show similar growth characteristics under various conditions (SK medium +/- lactate or nitrate).

While gut microbiome diversity decreased in both patients with ACLD and patients with sepsis, decreases in oral microbiome diversity were observed only in patients with ACLD (Extended Data Fig. 1a), with patients with AD and ACLF showing more drastic decreases in oral and gut microbiome diversity. Concurrently, microbial dysbiosis as measured by a dysbiosis index^34^ increased in both faeces and saliva in all ACLD groups (Extended Data Fig. 1b). Importantly, oral microbial dysbiosis seemed to occur at earlier disease severity stages with significant changes already present in cACLD compared with healthy controls (Extended Data Fig. 1b, Wilcoxon *P* = 0.0012). While the dysbiosis score reflects the dissimilarity of a given sample from the average healthy microbiome, the dysbiosis score distribution within healthy individuals reflects interindividual variations among this population for a given body site. Interestingly, dysbiosis scores were lower for healthy oral microbiomes than for healthy gut microbiomes (median_saliva_ = 0.55, median_faeces_ = 0.69; Extended Data Fig. 1b), suggesting that the oral microbiome across healthy individuals is more similar than their gut microbiomes.

Intra-patient oral and gut microbiome similarity was significantly increased in all ACLD groups (Fig. 1b, 1 − Bray–Curtis dissimilarity, Wilcoxon, adjusted *P* value < 0.001, Benjamini–Hochberg (BH) corrected). The number of shared species also increased in the ACLD groups (Fig. 1c, reference-based profiling), with *V. parvula, Veillonella atypica*, *Streptococcus salivarius* and *Streptococcus parasanguinis* being the most commonly shared species in paired saliva and faecal samples (Fig. 1d). Importantly, this increase in shared species was independent of the number of patients in each disease group (Extended Data Fig. 1c). *V. parvula* can ectopically colonize the gut during inflammation through nitrate respiration using the *narGHJI* operon^35^, and host-derived nitrate boosts *E. coli* growth^36^. We detected this *nar* operon in eight assembled Metagenomic Species Pangenomes (MSPs), including *E. coli*, *V. parvula* and *Veillonella dispar* (Extended Data Fig. 1d), which were among the most commonly shared oral–gut species in ACLD. Overall, this implicates the oral microbiome in ACLD progression, in which oral dysbiosis may precede changes in the gut.

### Co-occurring strains are detected in paired saliva and faecal samples in ACLD

**Fig. 2:**
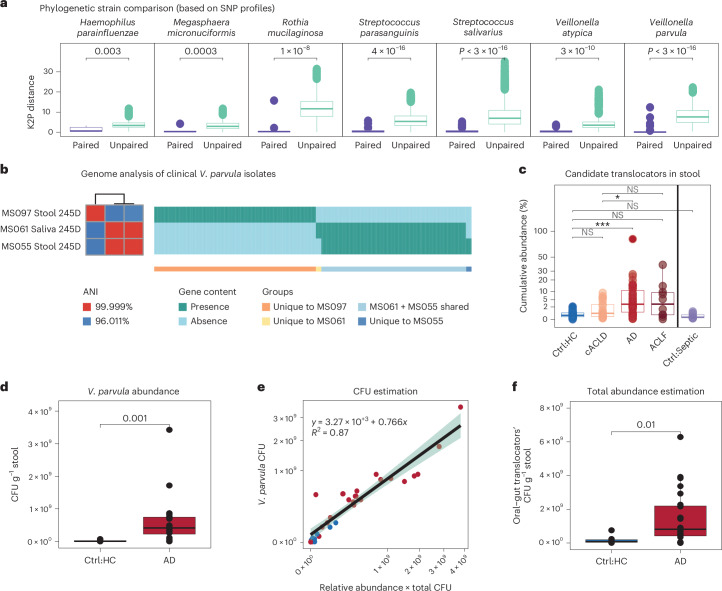
Identification of potential oral–gut translocators and their association with clinical ACLD parameters. **a**, Comparison of phylogenetic distance between strains from paired versus unpaired faecal and saliva samples. The *y*-axis shows K2P genetic distance estimates based on clade-specific marker genes (StrainPhlAn, two-sided Wilcoxon; SNP, single-nucleotide polymorphism). **b**, Genome analysis of clinical *V. parvula* isolates from paired faecal and saliva samples of a patient with AD (patient ID: 245D, AD). Genomes from three representative strains are shown, including phylogenetic relationship (left) based on genome-wide ANI and gene presence–absence patterns (right, PanPhlAn), in which genes shared by all three isolates were omitted (1,539 genes shared by all 3 isolates, 12 unique to MS061, 12 unique to MS055, 353 unique to MS097, 316 uniquely shared by MS061 and MS055). **c**, Cumulative relative faecal abundance of candidate translocators stratified by disease severity (two-sided Wilcoxon, *P* value adjusted with BH). **d**, Absolute abundance of *V. parvula* per disease group based on qPCR analysis (two-sided Wilcoxon). **e**, Comparison of quantitative *V. parvula* abundances (qPCR with *nifJ* primer, *y*-axis) and estimated *V. parvula* abundance based on total bacterial load multiplied by relative *V. parvula* abundance from MetaPhlAn (*x*-axis). A linear regression was fitted with *R* = 0.87. The green shading represents the 95% confidence interval of the linear fit, as generated by the geom_smooth() function in R. **f**, Absolute abundance of oral–gut translocators was estimated based on their cumulative relative abundance (MetaPhlAn) multiplied by total bacterial load (qPCR, universal 16S primer). A significant increase in the abundance of oral–gut translocators was observed in patients with AD (two-sided Wilcoxon). Adjusted *P* values: ***P < 0.001; **P = (0.001, 0.01]; *P = (0.01–0.05]; NS, *P* > 0.05. Source data

Strain-level profiling further supported the presence of oral–gut translocation in ACLD. As the gastro-intestinal tract provides a natural route for oral microorganisms to reach the gut, we focused our analysis on translocation from the mouth to the gut. First, we identified common members of the saliva microbiome (present in >20% of saliva samples) among the shared species (*n*_shared_ = 26; Fig. 1d and Supplementary Table 1), resulting in 9 candidate translocators. Two strategies were used to evaluate strain similarity: (1) phylogenetic distance between strains based on single-nucleotide polymorphisms (SNPs) in marker genes^37,38^ and (2) similarity of gene presence–absence patterns based on pangenome analysis^37,39^. These complementary strategies balance sequencing coverage requirements for SNP analysis with reference genome requirements for pangenome analysis. For all candidate translocator species except *Veillonella infantium*, at least one type of strain profile was available (Supplementary Table 1). For the remaining eight translocator species, oral and gut strains within the same individual were highly similar, while oral and gut strains in samples from different individuals showed significantly greater strain diversity (Fig. 2a and Extended Data Fig. 1e), providing computational evidence for bacterial oral–gut translocation.

Next, viable strains were isolated from patient samples based on the computationally identified translocation signals. Next-to-identical isolates for *V. parvula, V. dispar, Streptococcus gordonii* and *S. parasanguinis* were obtained from paired saliva and faecal samples with average nucleotide identities (ANI) > 99.98%. The subsequent follow-up investigation focused on the most frequent translocator in ACLD: *V. parvula*. *V. parvula* paired oral and faecal isolates showed an ANI of 99.999%, shared 99.4% of genes (MS061 and MS055, Fig. 2b) and exhibited highly similar growth patterns under various conditions (Extended Data Fig. 1f). Interestingly, an additional *V. parvula* strain was isolated from the same faecal sample with substantially higher strain heterogeneity compared with the next-to-identical isolate pair (MS061 and MS055), showing a genome-wide ANI of 96% and 353 unique genes (Fig. 2b). This strain heterogeneity motivated our subsequent investigation of common gene signatures among translocating strains.

### Increased oral–gut translocation in ACLD is associated with disease severity and intestinal barrier dysfunction

**Extended Data Fig. 2:**
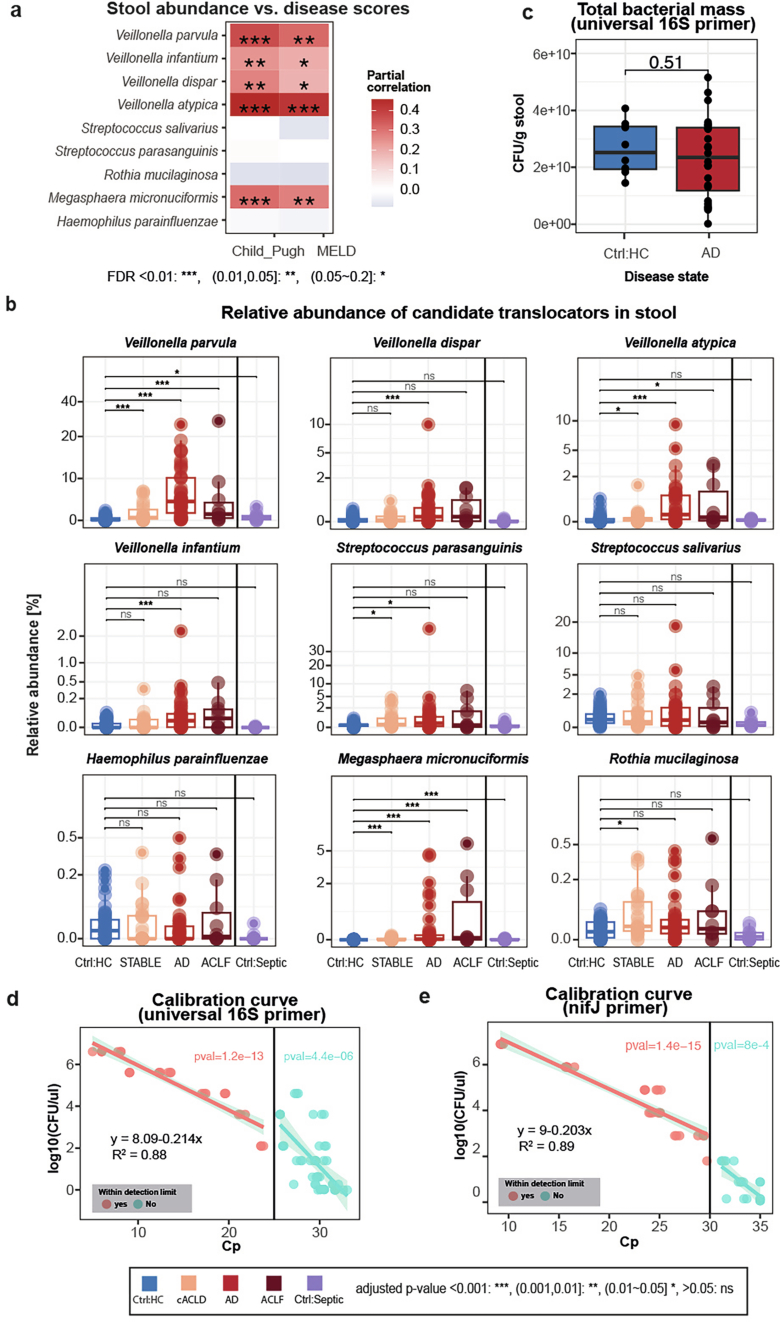
Increased oral-gut translocation in advanced chronic liver disease. (**a**) Correlation of faecal abundance of translocating species with Child-Pugh and MELD scores (partial correlation, Spearman, corrected for age, gender antibiotic usage, p-values were adjusted with BH, adjusted p-value < 0.01: ***, (0.01,0.05]: **, (0.05 ~ 0.2]: *) (**b**) Relative abundance of each oral-gut translocator in faeces per disease group (one-sided Wilcoxon, assessing whether total faecal abundance of translocators is increased in ACLD group, p-value adjusted with BH). (**c**) Total bacterial load estimation by qPCR showed comparable levels across disease groups (samples from 25 acutely decompensated ACLD (AD) and 8 healthy controls (Ctrl:HC) with on average (median) 23×10^9^ and 25×10^9^ colony-forming units (CFUs), respectively (Wilcoxon test). (**d**) Calibration curve based on universal 16S rRNA primer. Y-axis indicates CFU/ul for *Escherichia coli* and the x-axis shows the measured Cp value. Red points are within the detection limits (25 Cp) and the equation shows the fitted linear regression, p-value = 1.2e^−13^. (**e**) Analogous calibration curve for the *nifJ* primer targeting *V. parvula*. (Detection limits = 30 Cp and fitted linear regression with p-value = 1.4e^−15^).

The cumulative abundance of these oral–gut translocators significantly increased in the ACLD gut (Fig. 2c) with *V. parvula*, *V. atypica*, *S. parasanguinis* and *Megasphaera micronuciformis* enriched in cirrhosis (Extended Data Fig. 2b). While some of these species were also detected in healthy controls (for example, *S. salivarius* was present in >40% of paired healthy samples) and patients with sepsis, their faecal abundance was low (average total abundance: 0.4% and 0.2%, respectively). Notably, faecal abundances of *V. parvula*, *V. atypica*, *V. dispar*, *V. infantium* and *M. micronuciformis* showed positive correlations with ACLD severity (Extended Data Fig. 2a), including Child–Pugh and MELD scores, which are clinical measurements to evaluate the need for orthotopic liver transplantation and disease prognosis^40^. These analyses suggest that increasing oral–gut translocation may reflect ACLD progression.

These oral bacteria also increased in absolute abundances in the faeces of patients with AD. Comparable levels of total bacterial biomass were found in AD and healthy controls (Extended Data Fig. 2c), indicating a quantitative increase of oral bacteria in disease. We directly confirmed quantitative increases of *V. parvula* in AD (Fig. 2d). Importantly, *V. parvula* abundance estimation based on total microbial load and relative abundances agreed with the qPCR-based *V. parvula* measurements (Fig. 2e), confirming that species absolute abundances can be inferred based on relative abundance information in combination with total biomass estimation. Therefore, we also estimated overall absolute abundances for all oral–gut translocators (Fig. 2f), showing an increase of all identified oral–gut translocators in AD.

**Fig. 3:**
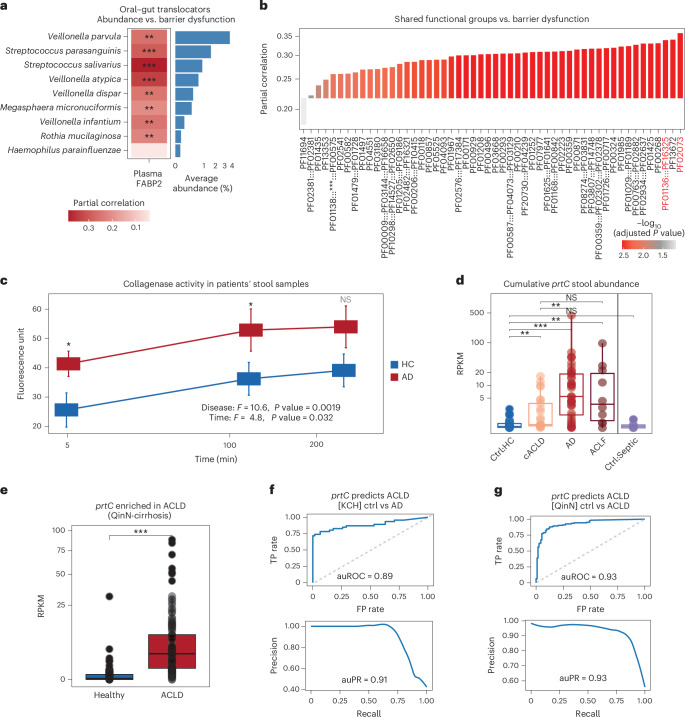
Functional analysis reveals putative microbial mechanisms resulting from oral–gut translocation that may be involved in intestinal barrier damage. **a**, Faecal abundance of oral–gut translocators was positively correlated with FABP2 levels (partial correlation; Spearman; corrected for age, gender and antibiotic usage; *P* values were adjusted using BH; adjusted *P* values: ***P < 0.01; **P = 0.01–0.05; *P = 0.05–0.2. Species were ordered by average faecal abundance in patients with AD (shown on the right). **b**, Faecal abundances of functional groups unique to translocators were positively correlated with FABP2 levels (partial correlation; Spearman; corrected for age, gender and antibiotic usage; *P* values were adjusted with BH). Proteinases are highlighted in red (*x*-axis), including thermophilic metalloprotease (M29, PF02073) and collagenase-like proteinase (prtC, PF01136:::PF16325). PF01138:::***:::PF00575 = PF03725:::PF03726:::PF01138:::PF03725:::PF00013”. **c**, Collagenase activity in faeces of patients with AD and healthy controls. The *x*-axis shows incubation time (min) and the *y*-axis shows fluorescence.Error bars indicate standard error (ANOVA; disease: *F*-statistic (*F*) = 10.6, *P* value = 0.0019; time: *F* = 4.8, *P* value = 0.032; one-sided *t*-test was used for pairwise comparison at each time point). Each group consists of 10 samples from different patients, and the collagenase activity of each sample is reported as the mean of three technical replicates. **d**, Cumulative *prtC* abundance in faeces compared across disease groups (two-sided Wilcoxon, *P* value adjusted with BH). **e**, Comparing total *prtC* faecal abundance (RPKM, *y*-axis) per disease group in a replication cohort (QinN-cirrhosis, *n*_healthy_ = 114, *n*_cirrhosis_ = 123, two-sided Wilcoxon). Species-level *prtC* homologues were identified using blastn on the corresponding gene catalogue (>95% identity, >90% coverage). At least one *prtC* gene was identified for each translocating species, and their cumulative abundance was significantly increased in the ACLD group (Wilcoxon, *P* value < 2.22 × 10^−16^, mean abundance increased 20.6-fold compared with the healthy group). **f**,**g**, ROC and PR curves for disease predictions (AD vs controls) based on *prtC* faecal abundance in our cohort (KCH, auROC = 0.89, auPR = 0.91) (**f**) and analogously for an independent replication cohort (ACLD vs controls, QinN, auROC = 0.93, auPR = 0.93) (**g**). Adjusted *P* values: ***P < 0.001; **P = (0.001, 0.01]; *P = (0.01–0.05]; NS, *P* > 0.05. Source data

**Extended Data Fig. 3:**
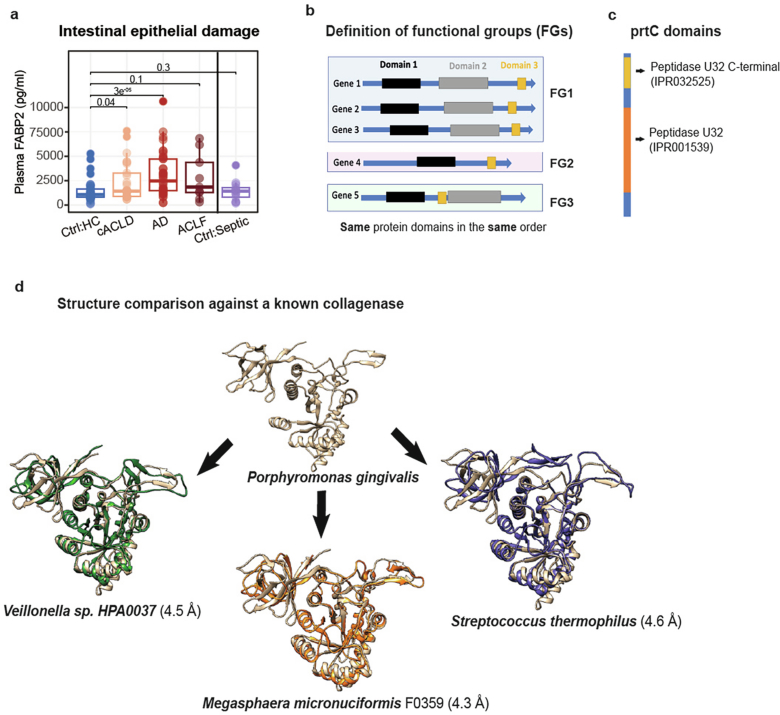
Identification of prtC gene uniquely shared among oral-gut translocators. (**a**) Fatty acid binding protein 2 (FABP2) levels (y-axis) were compared across different disease groups (Wilcoxon, alternative hypothesis: FABP2 reduced in Ctrl:HC, p-value adjusted with BH). (**b**) Schematic for the definition of functional groups (FGs), where protein encoding genes are grouped if they share the same functional domains in the same order. (**c**) Functional domain organisation of the prtC gene. (**d**) Structural comparison between *prtC* genes identified from ACLD patients and a well-characterised *prtC* gene from *Porphyromonas gingivitis* (P33437). The closest homologs in UniProt were identified using blastx Supplementary Table 2), structures were predicted by AlphaFold2 and then aligned against the *P. gingivalis prtC* gene. Structure differences are measured by root-mean-square-deviation (RMSD) in angstrom (Å).

Oral–gut translocation was associated with intestinal barrier dysfunction as measured by FABP2, a cytosolic fatty acid binding protein uniquely expressed in the small-intestinal epithelium^41–43^. Increased FABP2 levels in peripheral circulation indicate damage to the intestinal epithelium and increased epithelial permeability^44^. Plasma FABP2 levels increased in ACLD, in particular patients with AD (Extended Data Fig. 3a). Notably, faecal abundance of all identified oral–gut translocators was positively correlated with plasma FABP2 levels (Fig. 3a). Intestinal barrier impairment is hypothesized to drive cirrhosis-associated immune dysfunction, increasing the risk of infection^45,46^ by allowing bacteria and their products to enter systemic circulation and propagate hepatic inflammation via direct portal venous inflow^47,48^. Our results indicate a potential link between ectopic gut colonization of oral bacteria with ACLD progression and intestinal barrier impairment.

### ACLD intestinal barrier impairment is associated with a bacterial collagenase gene shared among oral–gut translocators

To identify aberrant host–microbial interactions involved in oral–gut translocation, we next looked for shared genes across translocating species that are typically absent in the gut microbiome. We grouped genes from the gene catalogue into functional groups (FGs, Extended Data Fig. 3b), in which genes encoding the same functional domains in the same order were grouped together. In total, 288 FGs were commonly shared by MSPs of oral–gut translocating species (>80% prevalence). To filter out housekeeping genes and FGs normally present in the gut, we excluded FGs from the core genomes of typical prevalent gut commensals (mean abundance >5% in healthy faecal samples), including *Prevotella copri*, *Bacteroides uniformis* and *Faecalibacterium prausnitzii*. After filtering, 52 FGs remained, potentially encoding mechanisms involved in oral–gut translocation. We next associated faecal abundances of these 52 FGs with plasma FABP2 levels (Fig. 3b). Two of the top FGs were involved in proteolytic activity, including a thermophilic metalloprotease (M29) and a peptidase with a U32 protein, which belongs to a proteinase family with collagen-degrading enzymatic activity. A well-studied case is protease C (PGN_RS02685, *prtC*) in *Porphyromonas gingivalis*, a pathogen associated with periodontal diseases, which degrades human type I collagen^49^, induces pro-inflammatory cytokines (IL-1a, IL-8 and TNF) and leads to tissue loss^50^. *PrtC* homologues shared by oral–gut translocators showed similar domain organization compared with the *P. gingivalis* collagenase-encoding gene (P33437), characterized by a peptidase U32 domain followed by an additional peptidase U32 C-terminal domain (Extended Data Fig. 3c). While sequence similarity was low (40%), their predicted structures were highly similar compared with the experimentally characterized *P. gingivalis* collagenase (root-mean-square deviation (RMSD) < 4.7 Å; Extended Data Fig. 3d), suggesting potential human type 1 collagen degradation ability.

Collagen degradation was also associated with ACLD pathophysiology. Collagenase activity in AD faecal samples was significantly higher compared with that in control samples (*P* < 0.0019, *F* = 10.6; Fig. 3c). Type I collagen is critical for structural tissue integrity^51,52^; therefore, higher collagenase activity suggests increased collagen degradation potentially leading to increased barrier dysfunction. Overall, this suggests that ectopic gut colonization by oral bacteria introduces microbiome functional shifts (that is, collagenase activity) that may contribute to disease-associated gut barrier impairment.

### Faecal *prtC* abundance is a robust ACLD biomarker

**Extended Data Fig. 4:**
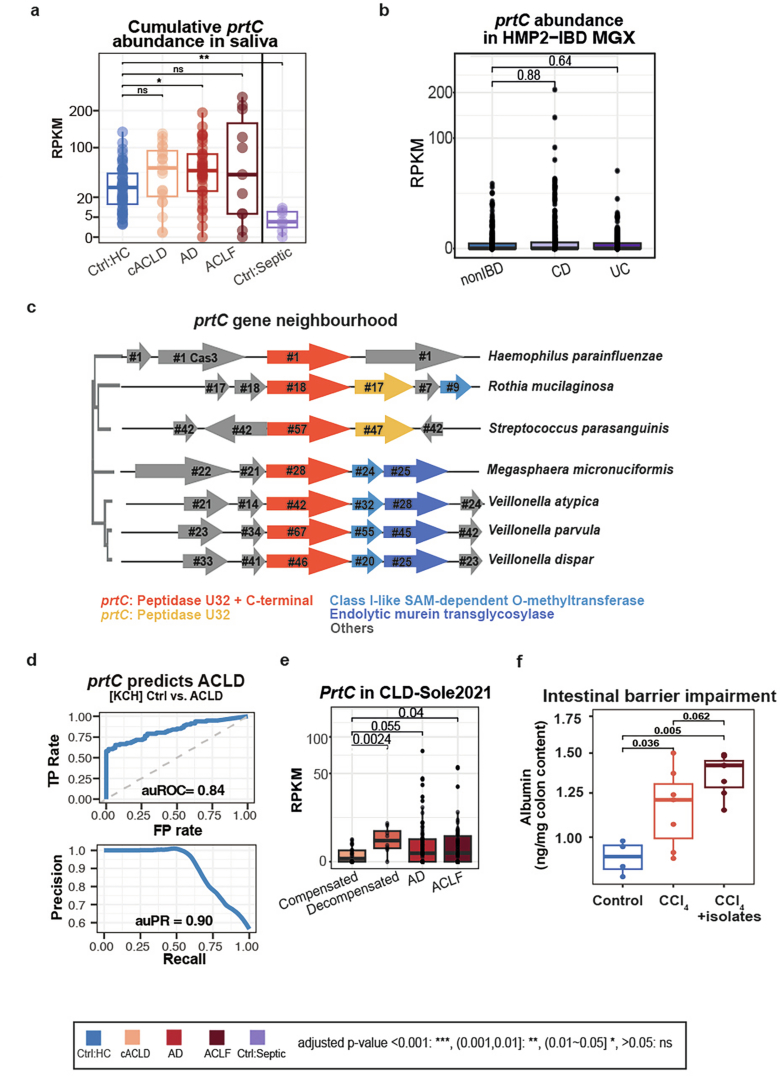
Faecal *prtC* abundance as a biomarker for advanced chronic liver disease. (**a**) Cumulative *prtC* abundance in saliva was compared across disease groups (Wilcoxon, p-value adjusted with BH). (**b**) *prtC* abundance per disease group in HMP2-IBD cohort (CD = Crohn’s disease, UC = ulcerative colitis, Wilcoxon). (**c**) *PrtC* gene neighbourhood in oral-gut translocators, including their phylogenetic relationship (tree on the left, based on *de-novo* assemblies). The number within each gene indicates the number of occurrences at this position relative to the center gene (*prtC* gene in orange, where the number indicates the number of assembled copies recovered from different samples). (**d**) ROC and PR curves of disease predictions (ACLD vs controls) based on prtC faecal abundance. (**e**) Faecal *prtC* abundance is significantly increased in severe cirrhosis (CLD-Sole2021, Wilcoxon). (**f**) Inoculation of human oral *Veillonella* and *Streptococcus spp*. increases intestinal barrier dysfunction in a mouse model of fibrosis. Intestinal permeability (measured by colon albumin level) compared across the non-treated group (control), CCl_4_-treated group (CCl_4_) and the CCl_4-_plus gavage group (CCl_4_+isolates) [one-sided Wilcoxon].

Total *prtC* abundance significantly increased in ACLD faecal and saliva samples, particularly for patients with AD (94.8-fold increase in faecal and 1.8-fold increase in saliva mean abundance in AD compared with healthy controls; Fig. 3d and Extended Data Fig. 4a). This cirrhosis-associated *prtC* gene signal was replicated in several independent cohorts (Supplementary Table 2). In the ACLD replication cohort (QinN-cirrhosis), we identified at least one *prtC* homologue at species level for all oral–gut translocators (>95% identity, >90% coverage). By contrast, in the inflammatory bowel disease (IBD) cohort (HMP2-IBD) only one *prtC* gene from *V. parvula* was present. Overall, *prtC* gene abundance was significantly increased in QinN-cirrhosis (Fig. 3e; 20.6-fold increase in cirrhosis) but not in HMP2-IBD (Extended Data Fig. 4b) or healthy populations (500FG^53^ and LifeLinesDEEP^54,55^; Supplementary Table 2). Together, these results suggest that oral–gut translocation in ACLD leads to ectopic gut colonization of collagen-degrading bacteria with potential to damage intestinal connective epithelial tissue leading to increased gut barrier dysfunction.

Using *prtC* cumulative abundances from all oral–gut translocators as a feature, we reliably distinguished controls (Ctrl:HC and Ctrl:Septic) from patients with ACLD (auROC = 0.84, auPR = 0.90; Extended Data Fig. 4d). Predictive power further increased for AD (auROC = 0.89, auPR = 0.91; Fig. 3f). These signals were confirmed in two ACLD replication cohorts (QinN-cirrhosis: auROC = 0.93, auPR = 0.93, Fig. 3g; CLD-Sole2021, Extended Data Fig. 4e). In previous studies, ACLD prediction models based on the abundance of 19 species combined with patient age achieved an auROC of only 0.86 in the QinN-cirrhosis cohort^12^. We also verified model specificity using two healthy cohorts, in which the model achieved 100% specificity in 500FG and 92.5% in LifeLinesDEEP. These analyses identify faecal *prtC* gene abundance as a strong and robust ACLD predictor.

### Clinical isolates of oral–gut translocators exacerbate intestinal barrier dysfunction and hepatic fibrosis in a CCl_4_ mouse model

**Fig. 4:**
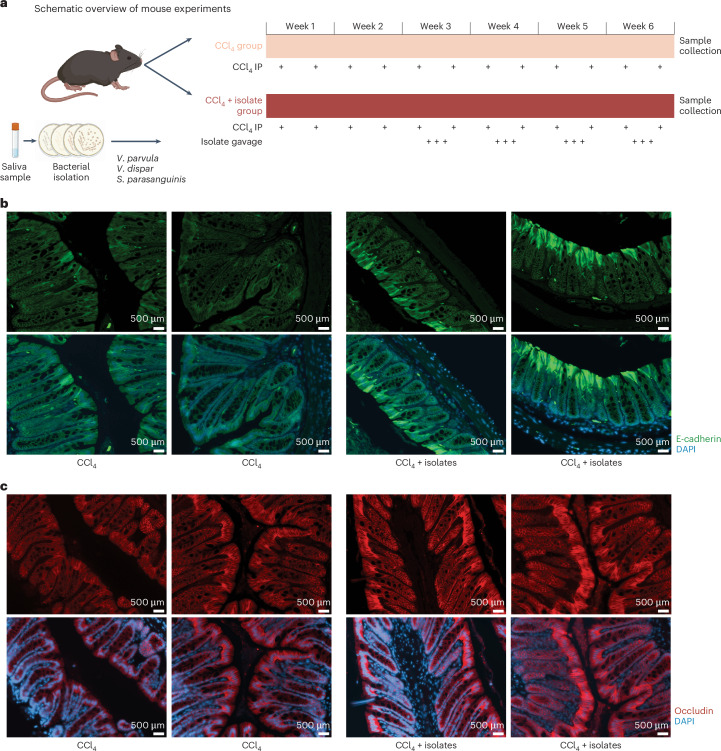
Clinical isolates linked to oral–gut translocation induce intestinal barrier dysfunction in a mouse model of fibrosis. **a**, Experimental overview: Strains associated with oral–gut translocation were isolated from patients’ saliva, including *V. parvula*, *V. dispar* and *S. parasanguinis* (Supplementary Table 1). C57BL/6 mice were treated IP with CCl_4_ twice a week for 6 weeks. After 2 weeks, one group of mice received oral gavage of patient saliva isolates 3× per week for 4 weeks (CCl_4_ + isolates). Mice were killed 2 days after the last CCl_4_ administration, and samples were collected for analysis. **b**, E-cadherin staining (green) highlighting adherens junctions along the epithelial lining. For each condition, two samples from two different mice were imaged. Additional images are available via Zenodo (Methods). **c**, Occludin staining (red) marking tight junctions between epithelial cells. Nuclei are counterstained with DAPI (blue) to provide structural context. Images are shown for both treatment groups (CCl_4_ and CCl_4_ + isolates) to illustrate differences in junctional protein localization. For each condition, two samples from two different mice were imaged. Additional images are available via Zenodo (Methods). Panel **a** created with BioRender.com.

Bacterial oral–gut translocators exacerbated gut barrier impairment in vivo. Mice were treated twice a week with CCl_4_ to induce hepatic fibrosis. After the first 2 weeks, one group of mice continued with this CCl_4_ treatment (CCl_4_ group) and the other group additionally received a cocktail of *prtC*-encoding oral isolates from patients with ACLD (*V. parvula*, *V. dispar* and *S. parasanguinis*; Supplementary Table 1) for three consecutive days per week (CCl_4_ + isolate group). At the end of week 6, mice were killed (Fig. 4a). While CCl_4_-treated mice showed a significant increase in colonic albumin compared with non-treated controls (*P* = 0.036 and *P* = 0.005, respectively), the highest albumin levels were observed for the CCl_4_ + isolate group (Extended Data Fig. 4f) indicating increased gut barrier disruption^56,57^. Immunofluorescent images provided additional evidence for disrupted epithelial junction architecture: tight junction proteins (E-cadherin and occludin) in the CCl_4_ + isolate group were relocated to the cytoplasm (Fig. 4b,c), indicating a loss of membrane localization^58^. These signals highlight a role for oral–gut translocating strains in disrupting gut barrier integrity.

**Fig. 5:**
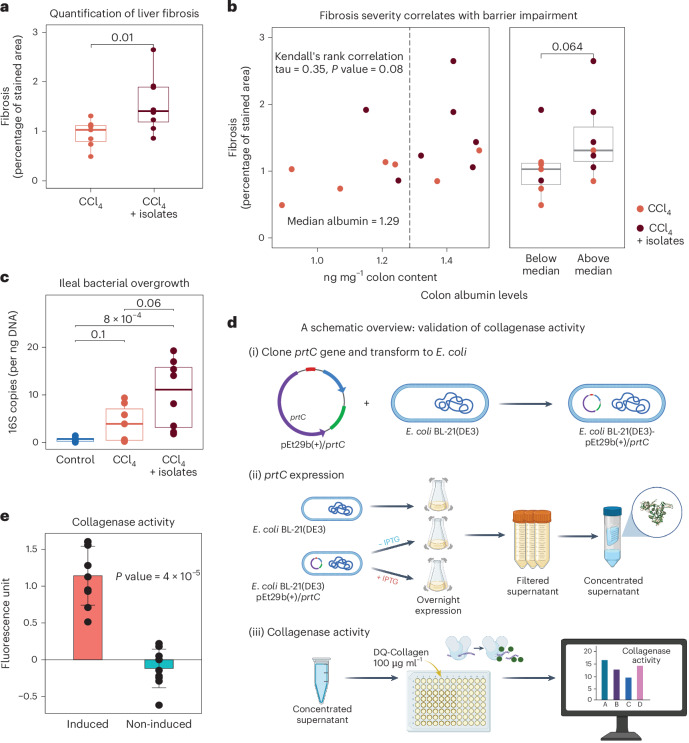
Oral clinical isolates with collagenase activity exacerbate liver fibrosis in mice. **a**, Liver fibrosis was quantified as the percentage of area stained by Sirius red. The *y*-axis refers to the estimated fraction of fibrous tissue for CCl_4_ or CCl_4_ + isolates (one-sided Wilcoxon). **b**, Fibrosis severity was positively correlated with intestinal barrier impairment (tau = 0.35, *P* value = 0.08, Kendall’s rank correlation). Left: the *x*-axis indicates albumin levels (ng mg^−1^ colonic content) and the *y*-axis represents the percentage of fibrosis in liver tissues. The dashed line shows the median albumin level across all samples, and colours indicate the different mouse groups. Right: samples were stratified into two groups based on albumin levels below or above the median. Fibrosis severity was compared between both groups (*P* value = 0.064, one-sided Wilcoxon). **c**, Comparison of ileal bacterial overgrowth among control, CCl₄-treated and CCl₄ + isolates-treated mice. The *y*-axis indicates bacterial load, quantified as 16S rRNA gene copies per nanogram of DNA (one-sided Wilcoxon). **d**, Schematic overview of the experimental validation of collagenase activity of the *V. parvula prtC* gene, including transformation of *E. coli* BL21(DE3) with pET29b(+)/*prtC* (i), *prtC* expression and supernatant concentration (ii) and collagenase activity measurements (iii). **e**, Comparison of collagenase activity of induced (+ IPTG) and non-induced (− IPTG) *E. coli* BL21(DE3)-pET29b(+)/*prtC* (two-sided Wilcoxon). Each condition was tested in three biological replicates, with two technical replicates per biological sample. The error bars represent the standard deviation of all data points within each condition. Panel **d** created with BioRender.com. Source data

**Extended Data Fig. 5:**
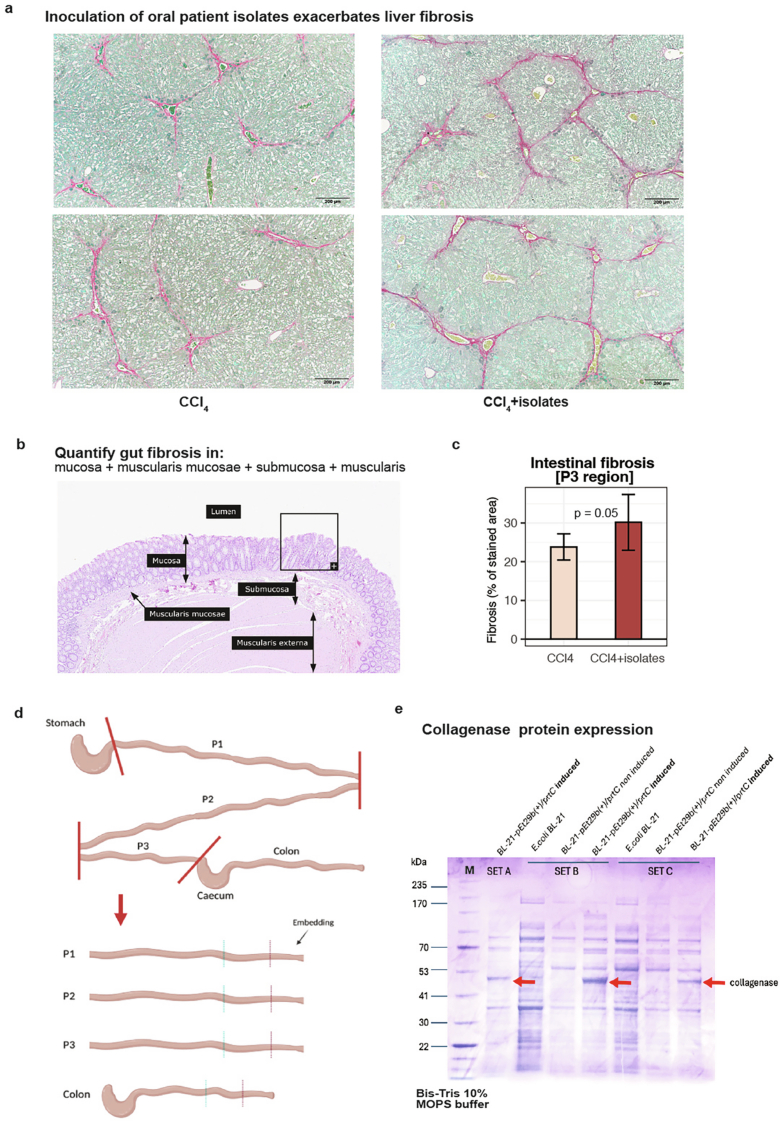
Experimental validation of *prtC* gene. (**a**) Inoculation of human oral *Veillonella* and *Streptococcus spp*. exacerbated hepatic fibrosis. Liver tissue was fixed, paraffin embedded and stained with Sirius red to detect fibrosis. Representative microscopic images at 10x magnification are shown, with collagen stained in red indicating regions of fibrosis. (**b**) Histological section of the gut stained with Sirius Red, showing labeled anatomical layers including lumen, mucosa, muscularis mucosae, submucosa, and muscularis extern. (**c**) Barplot showing a significantly higher percentage of Sirius Red-stained fibrotic area for the P3 region for CCl_4_+isolates compared to the CCl_4_ groups (p = 0.05, t-test). (**d**) Schematic representation of mouse gastrointestinal tract dissection showing isolated regions: stomach, duodenum (P1), jejunum (P2), ileum (P3), caecum and colon. (**e**) Recombinant collagenase protein expression in *E.coli* BL-21 (DE3) resulted in a band of the expected size (48 kDa), which was missing for the non-induced strains. M: Protein size marker; 10% SDS-PAGE stained with coomassie brilliant blue R250.

We also observed exacerbated hepatic and intestinal fibrosis and SIBO. Collagen deposition in the liver occurred in both the CCl_4_ and CCl_4_ + isolate group (Extended Data Fig. 5a), while the fraction of liver fibrotic tissue significantly increased in CCl_4_ + isolate mice (Fig. 5a) indicating an exacerbation of hepatic fibrosis. Furthermore, the degree of liver fibrosis correlated with colonic albumin levels (tau = 0.35, *P* value = 0.08, Kendall’s rank correlation; Fig. 5b), suggesting a link between gut barrier dysfunction and liver fibrosis exacerbation. Intestinal barrier impairment is recognized as a driver of hepatic fibrogenesis in ACLD and preserving gut integrity attenuates liver fibrosis^59^. Intestinal fibrosis was also quantified for the mucosa, muscularis mucosae, submucosa and muscularis regions (Extended Data Fig. 5b). Fibrosis-related disruption of mucosal architecture and barrier integrity is increasingly recognized as a key contributor to chronic liver disease development and progression^60^. Indeed, the CCl_4_ + isolate group showed significant increases in intestinal fibrosis (Extended Data Fig. 5c), suggesting a role of these clinical isolates in intestinal and liver fibrosis exacerbation. In addition, total bacterial load in the distal ileum (P3; Extended Data Fig. 5d) increased, revealing ileal bacterial overgrowth in CCl_4_ + isolate mice (Fig. 5c). Together, these results provide in vivo evidence for a causal role of oral bacteria in ACLD progression, including exacerbated gut barrier impairment and hepatic and intestinal fibrosis.

### Experimental validation of *V. parvula prtC* collagenase activity

Lastly, we confirmed collagenase activity of the bacterial *prtC* gene identified in oral–gut translocators focusing on *prtC*-encoding collagenase from *V. parvula*, the most frequent translocator. The gene was cloned into *E. coli* BL21(DE3) (Fig. 5d). Expression induction resulted in a distinct band corresponding to the expected molecular weight of the recombinant protein, which was absent in the supernatant of the non-induced strain (48 kDa; Extended Data Fig. 5e). Importantly, functional assays confirmed an active and functional collagenase protein (Fig. 5e).

Taken together, these results support the role of oral–gut translocation of bacterial strains, such as *prtC*-encoding *V. parvula*, and give mechanistic insights into how this may exacerbate ACLD disease progression, including gut barrier disruption through collagenase activity. This in turn may promote further microbial translocation along the gut–liver axis (leaky gut hypothesis) and the exacerbation of liver and intestinal fibrosis. Importantly, bacterial products in peripheral circulation, such as endotoxins^61–63^, are associated with immunological alterations and inflammation in ACLD^61^. Overall, intestinal barrier restoration and modulation of the oral microbiome may be promising avenues for preventing ACLD progression and the development of subsequent clinical complications, including hepatic decompensation and extrahepatic organ failure.

## Discussion

In this study, we investigated the role of oral–gut translocation in ACLD pathobiology. While increases in bacteria typical for the oral cavities in faecal samples of patients with ACLD were observed previously^12,23^, it was unknown whether these bacteria originate from the oral cavities (translocation), whether they ectopically colonize the gut (engraftment) and how they are involved in ACLD progression (disease contribution). Our results show that next-to-identical bacterial strains are present in paired saliva and faecal samples of patients with ACLD and that the identified oral–gut translocators increase in absolute abundances, providing evidence for gut engraftment. Importantly, we confirmed their disease involvement in vivo, where oral gavage with patient isolates exacerbated intestinal barrier impairment and gut and liver fibrosis in mice, providing mechanistic insights into how the oral microbiome may contribute to ACLD pathobiology.

Using microbiome strain and functional profiles, we bioinformatically identified putative microbial disease mechanisms, including a collagenase-like proteinase (*prtC*), which we linked to collagen degradation. *PrtC* homologues were uniquely encoded by oral–gut translocators but absent in commensal gut microorganisms. While structurally similar proteins are linked to epithelium damage in periodontal disease^64^, translocation-related consequences for the gut were unknown. Complementary evidence supports the relevance of this bacterial gene in ACLD, including (1) faecal *prtC* abundance, which showed strong positive correlations with markers of intestinal barrier damage (FABP2), (2) experimental validation of bacterial *prtC* collagenase activity using a recombinantly expressed *V. parvula prtC* gene and (3) higher collagenase activity in ACLD faecal samples. Previously, elevated collagenase activity was also reported for mouse faecal supernatant in the context of hepatocellular carcinoma (HCC) linking *Klebsiella pneumoniae* to carcinogenesis^65^. Our study provides clear evidence for a role of oral–gut translocation in collagenase increase and further motivates the investigation of the role of the oral microbiome in hepatocellular carcinoma. Importantly, we showed that faecal abundance of a single bacterial gene (that is, *prtC*) can serve as a robust biomarker to distinguish patients with ACLD and healthy individuals (auROC = 0.89, auPR = 0.91, sensitivity = 0.67, specificity = 0.89), putting it on par with current clinical non-invasive diagnostic methods, such as transient elastography (sensitivity = 0.61, specificity = 0.95 (ref. ^66^)) and magnetic resonance elastography (sensitivity = 0.86, specificity = 0.85 (ref. ^67^)).

Mice gavaged with oral patient isolates showed increased colon albumin levels, SIBO and endocytosis of tight junction proteins (E-cadherin and occludin), which moved from the basal compartment to the cytosol of gut epithelial cells. These in vivo findings are consistent with our observations in patients, in which oral bacteria increased in absolute abundance in ACLD faeces, which was associated with impaired gut barrier function (increased plasma FABP2 levels). Interestingly, mice gavaged with patient isolates showed other characteristics previously observed in patients with chronic intestinal inflammation, including gut fibrosis, which involves excessive extracellular matrix deposition and scarring that impairs intestinal function and barrier integrity by altering motility and mucosal architecture^68,69^. These gut changes facilitate SIBO and endotoxin translocation. Once they enter the portal vein, endotoxins activate Kupffer and hepatic stellate cells, driving hepatic inflammation and fibrogenesis^70^. Furthermore, the leaky gut hypothesis suggests that increased intestinal permeability^44^ leads to increases of bacterial products in systemic circulation, potentially leading to hepatic inflammation and subsequent disease progression^71,72^. Our study provides new evidence linking oral-originating microorganisms to a leaky gut in ACLD, implicating specific collagenase-degrading bacterial genes and strains from the oral cavity in intestinal barrier impairment and hepatic fibrosis progression.

There are limitations that apply to our study. Our findings are based on a cross-sectional, single-centre cohort located in the UK. We used five replication cohorts (USA, Europe and China) to ensure the applicability of our results to a wide spectrum of ethnicities and populations. Future work will include longitudinal sampling to measure sequential changes in oral–gut translocation and in response to therapy. Furthermore, parenteral antibiotic treatment is commonly required for hospitalized patients with ACLD and oral antibiotics are used as prophylaxis in patients with cACLD. Samples were collected as close to admission as possible (<48 h after commencement of antibiotic therapy) to limit antibiotic effects. While our healthy controls differed from the ACLD groups in age, antibiotic usage and proton-pump inhibitor exposure, the second positive disease control group, patients with sepsis, shared similar clinical characteristics (Table 1). Importantly, no abundance increase in oral–gut translocators was observed in patients with sepsis; instead, the increased similarity of saliva and faecal microbiome in sepsis was largely due to *E. coli* and *Enterococcus faecium* overgrowth. Both are common gut species that are absent from healthy oral cavities and thus not considered as oral–gut translocators in our analysis. Furthermore, while we implicated a combination of oral–gut translocating strains in disease exacerbation in mice, future work will be required to evaluate the effect of individual strains and genes, necessitating the development of genetic tools for these species. In addition, our mouse sample collection protocol did not allow for subsequent serum endotoxin quantification and fluorescein isothiocyanate (FITC)–dextran permeability assays and will be part of future studies to assess intestinal barrier integrity and systemic endotoxin exposure. Lastly, other potential confounding factors, such as oral hygiene, dental health and nutrition, may contribute to microbiome changes but were not recorded in this study.

In summary, we combined data-driven computational predictions from multi-omics data with in vitro and in vivo validation of host–microbial interactions to provide insights into the origin and consequences of oral–gut translocation in ACLD. Linking oral–gut translocation and the oral microbiome to ACLD pathobiology has the potential to facilitate the development of new therapeutic approaches. Re-establishing the commensal gut microbiome may also be a promising avenue to counteract ectopic gut colonization as already explored for Enterobacteriaceae infection^73^. Importantly, similar mechanisms may be at play in other chronic inflammatory diseases, as an enrichment of microorganisms typical of the oral cavities in the gut was also identified in IBD, colorectal cancer and rheumatoid arthritis^31^. Our study design and computational strategy provide a powerful framework to investigate and identify microbiome-derived mechanisms in these diseases.

## Methods

### Study participants and biological sampling

Patients were consecutively recruited at King’s College Hospital after admission to the ward or from the hepatology outpatient clinic. The study was granted ethics approval by the national research ethics committee (12/LO/1417) and local research and development department (KCH12-126) and performed conforming to the Declaration of Helsinki. Patient participants, or their family nominee as consultees in the case of lack of capacity, provided written informed consent within 48 h of presentation. Patients were managed according to standard evidence-based protocols and guidelines^74^. Patient and public involvement and engagement were undertaken with a patient advisory group that partnered with us to determine acceptability of the study; provided their perspective on study design, informational material and measures to minimize participation burden; and agreed on a dissemination plan of the findings.

Patient participants were stratified into and phenotyped according to clinically relevant groups based on the severity and time course of their underlying cirrhosis, degree of stability and hepatic decompensation, and presence and extent of hepatic and extrahepatic organ failure at the time of sampling. These groups were cACLD, AD and ACLF, with separately recruited healthy controls (Ctrl:HC) and patients with sepsis with no underlying ACLD as an additional control group (Ctrl:Septic). AD was defined as acute development of one or more major complications of cirrhosis, including ascites, hepatic encephalopathy, variceal haemorrhage and bacterial infection. ACLF was defined and graded according to the number of organ failures in concordance with criteria reported in the CANONIC study^75,76^. Main exclusion criteria included pregnancy, hepatic or non-hepatic malignancy, pre-existing immunosuppressive states, replicating HBV, HCV or HIV infection, and known IBD.

Demographic, clinical and biochemical metadata were collected at the time of biological sampling. Standard clinical composite scores used for risk stratification and prognostication included the Child–Pugh score^77^ and MELD^78^. For patients with sepsis without chronic liver disease (Ctrl:Septic), the diagnosis of sepsis was based on the Sepsis-3 criteria^79^ in which life-threatening organ dysfunction caused by a dysregulated host response to infection was evident, with organ dysfunction defined as an increase in the sequential (sepsis-related) organ failure assessment (SOFA) score of 2 points or more. The absence of chronic liver disease in this patient group was determined by a combined assessment of clinical history and biochemical and radiological parameters.

Healthy controls aged >18 years (*n* = 52) were recruited to establish reference values for the various assays performed. Exclusion criteria for healthy controls were body mass index <18 or >27; pregnancy or actively breastfeeding; personal history of thrombotic or liver disease; chronic medical conditions requiring regular primary or secondary care review and/or prescribed pharmacotherapies; or current use of anticoagulants, platelet function inhibitors or oral contraceptives.

### Plasma FABP2 quantification

Plasma samples for FABP2 profiling and quantification were obtained within 24 h of admission to hospital. Intestinal FABP2 (refs. ^42,44^) was quantified to serve as a gut-specific marker of intestinal barrier integrity, to assess whether these differentiated at the different stages of cirrhosis and in the Ctrl:HC cohort to define whether physiological or basal levels were detectable. FABP2 was quantified using the human FABP2/I-FABP Quantikine enzyme-linked immunosorbent assay (ELISA) kit (R&D Systems). All assays were conducted according to the manufacturers’ instructions. Optical densities were measured with a FLUOstar Omega absorbance microplate reader.

### Faecal and saliva sample acquisition

Faecal samples were obtained within 48 h of admission to hospital and collected into non-treated sterile universal tubes (Alpha Laboratories) without any additives. Faecal samples were kept at 4 °C without any preservative and were homogenized within 2 h and pre-weighed into 200-mg aliquots in Fastprep tubes (MP Biomedicals). Saliva samples were obtained within 48 h of admission and collected into non-treated sterile universal tubes (Alpha Laboratories) without any additives. A controlled passive ‘drool’ was performed by the study participant into a universal container repeatedly until at least 6 ml of saliva was obtained. For patients who were intubated for mechanical ventilation, oropharyngeal suctioning of accumulating oral secretions was performed. Saliva samples were kept at 4 °C without any preservative and within 2 h were homogenized and measured into 1-ml aliquots using sterile wide-bore pipettes in Fastprep tubes (MP Biomedicals), which were then centrifuged at 17,000 *g* for 10 min. The saliva supernatant was removed and stored separately from the remaining pellet. Faecal and saliva samples were stored at −80 °C for subsequent DNA extraction and metabolite measurements.

### Metagenomic data generation for saliva and faecal samples

Metagenomic data of SRA Project ID PRJEB52891 were generated as part of a previous study^80^. Briefly, microbial DNA was extracted from stored faecal and saliva pelleted samples using a 2-day protocol adapted from the International Human Microbiome Standards^81,82^. A 200-mg pre-weighed and homogenized aliquot was used for faeces, while for saliva, a post-centrifugation pellet was used. For the processing of additional healthy control samples (project ID: PRJNA1307628), sample collection and storage were identical to those of the initial cohort samples. DNA for the additional faecal samples was extracted with the AllPrep PowerFecal DNA/RNA Kit Qiagen (kit catalogue number 80244), and for saliva samples, the extraction kit DNeasy PowerSoil Pro Kit (Qiagen, 47014) was used according to the manufacturer’s instructions. DNA was subsequently sequenced (Illumina NovoSeq, paired-end mode, read length 2 × 150 bp) with a targeted sequencing depth of 25 Gbp for both saliva and faecal samples. Reads retained after quality control were uploaded to the Sequence Read Archive (SRA).

### Quantification of absolute bacterial abundances using qPCR

To quantify total bacterial biomass and *V. parvula* absolute abundances, we performed qPCR assays following the protocol of previous studies^35,83^. Briefly, we first conducted serial dilutions of *E. coli* (strain BL21(DE3)) culture grown in Gifu Anaerobic Broth, modified agar (mGAM) medium and enumerated colony-forming units (CFU) for each of the dilutions. Next, DNA isolation was performed using 6 ml of *E. coli* culture applying phenol-chloroform-isoamylalcohol as described in a previous study^84^. A universal 16S rRNA primer^83^ targeting the V9 region (forward: AGAGTTTGATYMTGGCTCAG; reverse: TACGGYTACCTTGTTACG ACT) was used, and 2 μl of DNA was added to the mix of 2 μl of nuclease-free water, 0.5 μl of forward and reverse primers (10 μM) and 5 μl of 2 × SensiFast SYBR mix (BioCat). The assay was run according to the following set-up: initial denaturation for 2 min at 95 °C, 40 cycles of 3-step cycling at 95 °C for 15 s, 53 °C for 15 s and 72 °C for 15 s followed by melting at 95 °C for 30 s, 53 °C for 30 s and 60 °C for 1 s with final cooling at 40 °C for 30 s. The same procedure was repeated for the *V. parvula* patient isolate with *nifJ* gene-specific primers introduced in a previous study^35^ (forward: TGGTGACCACCAAGACGTAA; reverse: ACAGCATCCATGTCAACCAA). Based on the computed crossing point (Cp) values, calibration curves were established as described in a previous study^85^ to set ratios between cycle threshold (Ct) values and calculated CFUs further enabling estimations of CFU per gram faeces (Extended Data Fig. 2d,e). Next, patient samples were processed using the same qPCR set-up and master mix with both V9 region 16S and *V. parvula-*specific *nifJ* primers and compared with the calibration curve.

### Metagenomic data analysis

The metaGEAR pipeline was used for metagenomic quality control, reference-based microbial profiling and gene-centric analyses (https://github.com/schirmer-lab/metagear-pipeline). Briefly, raw metagenomic reads went through the following quality control pipeline to remove (1) sequencing adaptors, (2) low-quality reads and (3) human host contaminations. First, we applied trim_galore^86^ for adaptor removal using the parameters ‘--paired --phred33 --quality 0 --stringency 5 --length 10’. Then, we applied KneadData (https://github.com/biobakery/kneaddata) to remove low-quality reads and host contaminations. Low-quality reads and adaptors were removed using the parameters ‘--trimmomatic-options ‘SLIDINGWINDOW:4:15 MINLEN:50’’. Human reads were removed by mapping against the human reference genome (hg38). In addition, tandem repeats were removed using the default ‘trf’ option. The ‘--reorder’ flag was applied to match quality-controlled forward and reverse reads.

For reference-based microbial profiling and strain comparison, we first applied MetaPhlAn3 (ref. ^37^) to profile the microbial community based on clade-specific marker genes. Strain-level profiles were then generated for the species of interest based on (1) SNPs in the marker genes using StrainPhlAn3 (ref. ^37^) and (2) presence–absence profiling of gene families using PanPhlAn3 (ref. ^37^).

Assembly-based analyses for generating metagenomic assembled pangenomes included de novo assembly using MEGAHIT with default parameters^87^ for each sample. Then, protein-coding genes were predicted using Prodigal with the ‘-p meta’ flag^88,89^. Incomplete genes were subsequently filtered with the ‘partial==00’ flag. Afterwards, genes from different samples were merged and clustered to generate a gene catalogue using cd-hit-est^90^ with parameters ‘-aS 0.9 -aL 0.9 -c 0.95’ to group genes with sequence identity >95% and coverage >90%. Protein sequences were extracted for the representative sequences of each gene family and further grouped into a protein catalogue using cd-hit^90,91^ with parameters ‘-c 0.9 -aL 0.8 -aS 0.8’ to group proteins with sequence identity >90% and coverage >80%. Abundance profiles for the clustered genes were generated using CoverM^92^ contig (methods count and reads per kilobase per million mapped reads (RPKM)) using parameters ‘--min-read-percent-identity 95 --min-read-aligned-percent 75 --min-covered-fraction 20’.

The protein catalogue was annotated with interproscan v5.47-82.0 (refs. ^93,94^) using the parameter ‘-appl Pfam’ to get domain-level annotation. Functional group annotation was extracted for each representative sequence of the protein families by combining Pfam domain annotations according to their order for each protein. Afterwards, reads from each sample were mapped against the gene catalogue to obtain the respective abundance profiles. The MSPs were generated with MSPminer^95^ with its default parameters. Taxonomy annotation for each MSP was predicted based on gtdb-tk (v2.3.0)^96^ on its pangenomes, and the abundance of each MSP was profiled by the median abundance of its core genes in each sample.

### Integration of reference- and assembly-based results

Many MSPs lacked species-level taxonomy annotations based on gtdb-tk annotations. Therefore, we introduced a new taxonomy annotation approach for MSPs that additionally integrates information from reference-based profiles. This approach is based on the assumption that each MSP will have a similar abundance profile as its corresponding referenced-based counterpart. Therefore, we inferred MSP taxonomic information based on abundance correlations with reference-based abundance profiles (MetaPhlAn3). For each MSP, the species from the MetaPhlAn profile with the best abundance correlation was assigned as its taxonomic annotation.

### Definition of dysbiosis score

We adapted the dysbiosis score introduced by a previous study^34^ to quantify the deviation of a given patient sample from the healthy control group. For our study, we calculated the dysbiosis separately for faecal and saliva samples. Specifically, for each body site, the dysbiosis score *D* for sample *x* is defined as the median Bray–Curtis distance to the samples from the healthy control group:

$$D(x)={\mathrm{median}}_{s\in \mathrm{HC}}\{{\mathrm{dist}}_{\mathrm{Bray}-\mathrm{Curtis}}(x,s)\}$$

### Identification of candidate oral–gut translocators

First, we identified co-occurring species within each sample pair. For this, species were required to be present with a relative abundance of >0.1% in both sample types. Species were defined as candidate oral–gut translocators if they (1) frequently co-occurred in paired faecal and saliva samples (detected in at least five paired samples) and (2) were common members of the healthy oral microbiome (>1% abundance and detected in >20% of saliva samples). Overall, the number of co-occurring species was 26 (details in Supplementary Table 1). Among these, nine species were commonly detected in the oral samples, forming the final list of our candidate oral–gut translocators for the subsequent analyses. For each candidate translocator, we then compared the strain-level similarity between paired and unpaired samples using two different strategies: Phylogenetic similarity was quantified with the Kimura 2-parameter (K2P) genetic distance based on marker gene alignment generated with StrainPhlAn3 (ref. ^37^). The function distmat (https://www.bioinformatics.nl/cgi-bin/emboss/help/distmat) was used, which calculates the evolutionary distance between every pair of sequences in a multiple sequence alignment. Distances are expressed in terms of the number of substitutions per 100 bases. In addition, gene content similarity was compared, which was measured by the binary distance between the gene content of strains inferred with PanPhlAn3 (ref. ^37^).

### Associations of bacterial species with disease severity and plasma barrier dysfunction

The faecal abundance of oral–gut translocators was analysed for associations with clinical indices, including disease severity (Child–Pugh and MELD scores) and barrier dysfunction (plasma FABP2). We used the pcor.test function in R to calculate partial correlations and corresponding *P* values between each species and each clinical index. Associations were computed using a rank-based Spearman method, with age, gender and antibiotic usage included as covariates. *P* values were calculated using asymptotic *t* approximation (with default options in the pcor.test function). The detection limit for MetaPhlAn was set at 0.01%, with abundances below this threshold set to zero. BH-adjusted *P* values were calculated separately for each clinical index, and results were visualized as a heatmap in which colours indicate partial correlation and asterisks denote significance levels (***P < 0.01; **P = 0.01–0.05; *P = 0.05–0.2).

### Statistics and reproducibility

This is a non-interventional, cross-sectional, prospective cohort study. No predefined sample size was determined, and no data were excluded from the analyses. Our sample sizes are comparable to those reported in previous publications^26,80,97^. Data collection and analysis were not performed blind to the conditions of the experiments.

#### Statistical analyses involving the five disease subgroups

For these analyses, the healthy control group, Ctrl:HC, was compared with each of the disease subgroups, including cACLD, AD, ACLF and Ctrl:Septic. In addition, the cACLD group was compared with AD and ACLF to evaluate differences involved in disease progression. This resulted in six statistical tests in total. All *P* values were correct for multiple testing (BH), and adjusted *P* values were reported (Figs. 1b,c, 2c and 3d and Extended Data Fig. 1a). For a subset of supplementary figures, the primary focus was on identifying changes between healthy controls (Ctrl:HC) and disease subgroups; here only four statistical comparisons were performed and corrected for multiple testing (Extended Data Figs. 1b, 2b, 3a and 4a).

#### Associations between oral–gut translocators and clinical indices

To test for associations between the nine species identified as potential oral–gut translocators with Child–Pugh, MELD scores and plasma FABP2 levels, respectively, nine statistical tests were performed for each index. All *P* values were corrected for multiple testing (BH) and adjusted *P* values are reported (Extended Data Figs. 2a and 3a).

All *P* values mentioned above are calculated using two-side Wilcoxon tests.

### Strain isolation

Saliva and faecal strains were isolated on SK agar plates (as used in previous work^98^) supplemented with vancomycin (Vc; 7.5 μg μl^−1^), chloramphenicol (Cm; 0.5 μg μl^−1^) and erythromycin (Ery; 14.6 μg μl^−1^), in addition to mGAM agar plates (HiMedia M2079). SK (Vc, Cm and Ery) and mGAM agar plates (1.5% agar, Fisher Scientific, 10572775) were prepared. For plates supplemented with Vc, Cm and Ery, antibiotics were added during the plate preparation. All plates were transferred to an anaerobic chamber under anaerobic conditions (5% H_2_, 10% CO_2_ and 85% N_2_) to be pre-reduced for 24 h before plating. Frozen saliva and faecal samples were thawed in the anaerobic chamber and serial dilution was realized, in which 100 μl of diluted samples was dispensed and spread on the plates. The plates were incubated for up to 4 days at 37 °C in the anaerobic chamber for colony growth.

After each day, strain imaging and colony picking were performed for further isolation. The colony picking was based on the identification of morphologically unique colonies including area perimeter, circularity, convexity, colour and consistency. Colonies were stricken on the equivalent media from which they were picked and incubated at 37 °C in the anaerobic chamber for colony growth.

### Bacterial strains and growth conditions

Supplementary Table 1 lists the bacterial strains used in this study. Strains were grown at 37 °C in an anaerobic chamber (Whitley M45, Meintrup DWS Laborgeräte) at an atmosphere of 5% H_2_, 5% CO_2_ and 90% N_2_. MS082 was cultivated on mGAM (Himedia) agar plates. Strains MS055, MS061, MS072, MS097, MS107 and MS164 were cultivated in SK broth or on agar plates (Difco tryptone 10 g l^−1^, yeast extract 10 g l^−1^, NaCl 2 g l^−1^, Na_2_HPO_4_ 0.4 g l^−1^)^35^.

### Growth curve characteristics of *Veillonella* strains

Strains MS055 and MS061 were grown in SK broth as detailed under the ‘Bacterial strains and growth conditions’ section, and in SK medium supplemented with 50 mM DL-lactate (Sigma-Aldrich) or 50 mM potassium nitrate (Sigma-Aldrich), respectively. In brief, overnight cultures of MS055 and MS061 in SK medium were diluted into fresh medium for log-phase growth and used to inoculate the main culture of 6 ml medium (start optical density at 600 nm (OD_600_) 0.05) of SK medium only, and SK medium with 50 mM lactate or nitrate, respectively. Growth of strains was monitored every 2 h using a spectrophotometer (CO8000, Biowave). The results represent three independent experiments and are presented as means with standard errors of the means.

### Gene neighbourhood visualization from de novo assemblies

For each gene family of interest, we retrieved all assembled contigs containing this gene. Then, we aligned the contigs centred to the gene of interest taking strand information into account. The gene families in the same relative position to the centre gene were retrieved, and the most prevalent gene family at each relative position was visualized including the number of observations of that gene.

### Identification of *prtC* homologues in MSPs

Each candidate oral–gut translocator species was first matched to their corresponding MSPs based on gtdb-tk taxonomic annotations of the assembled MSPs and linear correlations of the respective MetaPhlAn and MSP abundances (for further details, see section ‘Integration of reference- and assembly-based results’). We were able to assign MSPs for seven out of nine oral–gut translocators. FG annotations for each gene family in the MSPs were extracted, and 288 FGs were found to be commonly shared among MSPs associated with oral–gut translocation (>80% prevalence). Next, we excluded functional groups that represent functions commonly found in the gut, by comparing these 288 FGs to those present in the core genomes of prevalent gut commensals. For this comparison, we selected MSPs with a mean abundance greater than 5% across all healthy faecal samples, including *P. copri*, *B. uniformis* and *F. prausnitzii*. FGs were filtered if they were also present in these common healthy gut microorganisms, and this filtering step reduced the number of remaining FGs to 52, which may represent bacterial functions that are essential for oral–gut translocation and/or result in aberrant host–microbial interactions in disease. Cumulative faecal abundances for these 52 FGs were calculated by summing over all matched gene families in oral–gut translocators. Subsequently, we further prioritized these FGs according to the correlations between their faecal abundance and plasma FABP2 levels. For this, the pcor.test function in R (rank-based Spearman) was used to calculate partial correlations with age, gender and antibiotic usage included as covariates. *P* values were corrected for multiple testing with BH.

### Predicting ACLD based on faecal *prtC* gene abundance

First, the cumulative abundance of *prtC* genes encoded by oral–gut translocators was inferred for each faecal sample and represented as a single value with the detection limit set to 0.5 RPKM. These values were then used to predict disease status: any sample with a value above this threshold was predicted as ‘ACLD’, and samples below this value as ‘control’. The confusion matrix, specificity and sensitivity are reported in Supplementary Table 2. The corresponding receiver operating characteristic (ROC) and precision–recall (PR) curves were generated using the cumulative *prtC* abundance as the predictor and actual disease status (based on the clinical metadata) as the ground truth (1 for ACLD, 0 for controls)

### Verification of the *prtC* signals in CLD-Sole2021 cohort

The cohort of a previous study^11^ (CLD-Sole2021) contained metagenomic data with single-end reads based on ion proton sequencing. Due to the shorter read length and lower read quality, we did not assemble this dataset but used a mapping-based strategy (bowtie2) against the gene catalogue generated from our cohort. RPKM values are calculated from reads mapped to the *prtC* genes.

### Structure comparisons between *prtC* genes and a known bacterial collagenase

We applied blastx to search the UniProt database using gene sequences of the *prtC* genes identified in our cohort (Supplementary Table 2). The best hit for each query sequence was taken for further analysis, in which the predicted protein structure was downloaded as .pdb format and was aligned against the structure of a well-characterized bacterial collagenase (P33437). In cases in which multiple queries matched the same protein from the UniProt database, we took only one matched protein for downstream analysis. For example, the *prtC* genes from *V. parvula*, *V. atypica* and *V. dispar* were all mapped to *Veillonella* sp. HPA0037; therefore, we used the predicted *prtC* structure from *Veillonella* sp. HPA0037 for the alignment to represent these three *prtC* genes. To quantify the structural similarity, the RMSD was calculated for each structure alignment.

### Experimental procedures in animals

In vivo experimental procedures were performed in 10–12-week-old male C57/Bl6 mice at the Disease Modelling Unit (University of East Anglia, UK). Experiments were approved by the Animal Welfare and Ethical Review Body (University of East Anglia, Norwich, UK) and the UK Home Office (project licence to Beraza: PP9417531, protocol 6). All procedures were carried out following the guidelines of the National Academy of Sciences (National Institutes of Health, publication 86-23, revised 1985) and were performed within the provisions of the Animals (Scientific Procedures) Act 1986. All animals were provided with free access to food (EURodent Diet 22%) and water. The animals were randomly assigned to cages and to the various experimental groups. No animals or data were excluded.

### Induction of hepatic fibrosis in vivo

Mice were treated with CCl_4_ (1 ml kg^−1^) that was administered intraperitoneally (IP) twice per week for a total of 6 weeks. One group of mice received 200 μl of a bacterial cocktail (10^9^ CFU ml^−1^) composed of *V. parvula* (isolates MS061, MS107 and MS164), *V. dispar* (isolate MS072) and *S. parasanguinis* (isolate MS082) by oral gavage 2 weeks after the initiation of CCl_4_ treatment. The strain cocktail was administered for three consecutive days each week for 4 weeks (week 3 to week 6 of treatment). All mice were killed 2 days after the last administration of CCl_4_ (Fig. 4a).

### Quantification of fibrosis severity in mice

Liver tissues were fixed in 10% neutral buffered formalin (Sigma–HT501128-4L), embedded in paraffin and sectioned. Liver sections were dewaxed, hydrated and stained with Sirius red to stain collagen and detect fibrosis. Slides were imaged on a BX53 upright microscope (Olympus) with an Olympus DP74 colour camera and a pT100 LED transmitted light source (CoolLED). For quantification of the deposition of collagen in the liver, a total of 6–10 fields of view per sample were imaged and analysed using open-source FIJI software^99^. Images were split into three channels using the Colour tool, and the green channel was selected for quantification. A consistent threshold was applied across all images. Fibrosis was represented as the percentage of stained area relative to the total area per field^100^.

### Quantification of gut barrier dysfunction in mice

Frozen faecal material from the large intestine was weighed and diluted in a 1:15 weight-to-volume ratio using extraction buffer (50 mM Tris HCl, 150 mM NaCl, 0.1% SDS, 2 mM EDTA (pH 8.0)). Samples were homogenized 3 times at 6 m s^−1^ for 40 s, and homogenates were centrifuged at 13,523 *g* (12,000 rpm) at 4 °C for 10 min. Supernatants were collected and assessed for albumin levels. Albumin levels were quantified using the DY1455 human albumin Duoset ELISA from R&D Systems according to the manufacturer’s instructions. Results were quantified using a FluoStar Optima plate reader. The mouse gastro-intestinal tract was dissected into anatomically defined regions, and the terminal portions of the ileum and colon were fixed and embedded in paraffin for immunohistological analysis: slides were mounted with a 4′,6-diamidino-2-phenylindole (DAPI)-mounting solution (Vector Laboratories H-1200) to stain cell nuclei. Fluorescence microscopic imaging was performed using an AxioImager M2 (Zeiss) with the AxioCam mRM monochrome camera and standard light source and filter sets supplied.

### Preparation of faecal water

Frozen faecal samples from patients with AD (*n* = 10) and healthy individuals (*n* = 10) were thawed on ice. A 0.3-g aliquot from each sample was suspended in 2 ml of 1× phosphate-buffered saline and homogenized thoroughly. The homogenates were centrifuged at 5,000 *g* for 30 min at 4 °C. The resulting supernatants were collected and subjected to two additional centrifugation steps at 5,000 *g* for 20 min and 10 min, respectively, both at 4 °C. Supernatants were collected and kept on ice after each step for downstream analysis.

### *E. coli* BL21(DE3) pET29b(+)/*prtC* transformation

The *prtC* gene sequence from *V. parvula* (isolate MS055) was synthetized and cloned in pEt29b(+) by TwistBioscience. Competent *E. coli* BL21 was transformed by heat shock with the pET-29b(+)/*prtC* vector and plated on Luria broth agar media supplemented with kanamycin (25 μg ml^−1^) and incubated for 24 h at 37 °C.

### Recombinant protein expression and preparation

*E. coli* BL21(DE3) and BL21(DE3) harbouring the pET-29b(+)/*prtC* plasmid were cultured in 100 ml Luria broth at 25 °C until reaching an optical density (OD₆₀₀) of 0.8. Protein expression in BL21(DE3) pET-29b(+)/*prtC* was induced with 0.1 M isopropyl β-D-1-thiogalactopyranoside (IPTG), followed by incubation at 25 °C for 18 h. Induced and non-induced cultures were centrifuged at 5,000 *g* for 20 min at 4 °C. The resulting supernatants were transferred to Amicon Ultra Centrifugal Filter tubes and centrifuged again at 5,000 *g* for 10 min at 4 °C.

### Collagenase activity in faecal water: collagen degradation assay

Collagen degradation was assessed using the EnzChek Gelatinase/Collagenase Assay Kit (Thermo Fisher Scientific). DQ-collagen I was added to concentrated supernatants or faecal water, a media-only control and a positive control (*Clostridium histolyticum*’s collagenase) at a final concentration of 100 µg ml^−1^. Samples were incubated overnight at 37 °C. Fluorescence was measured at an excitation wavelength of 495 nm and emission at 515 nm. The fluorescence intensity was directly proportional to the extent of collagen degradation.

### Reporting summary

Further information on research design is available in the Nature Portfolio Reporting Summary linked to this article.

## Supplementary information

Supplementary Information The original gel image for Extended Data Fig. 5e.

Reporting Summary

Supplementary Table 1 Sample overview, bacterial strains used in this study and oral-gut translocation events details.

Supplementary Table 2 Metadata, clinical measurements, microbial profiles and results for prtC-based prediction.

Supplementary Table 3 Measurements from CCl₄ mouse experiments.

## Source data

Source Data Fig. 1 Statistical source data.

Source Data Fig. 2 Statistical source data.

Source Data Fig. 3 Statistical source data.

Source Data Fig. 5 Statistical source data.

## Data Availability

Metagenomic reads are available through SRA Project ID PRJEB52891 (ref. ^80^) and PRJNA1307628. Processed metagenomic profiles, including MetaPhlAn3 taxonomic profiles, MSP abundances and MSP taxonomy annotation, are included in Supplementary Table 2. All additional data related to this work are also included in Supplementary Table 2, including clinical metadata, FABP2 measurements and disease scores. Raw images of immunofluorescence imaging and Sirius red staining can be found via Zenodo at 10.5281/zenodo.17523438 (ref. ^101^) and 10.5281/zenodo.17523671 (ref. ^102^). Source data are provided with this paper.

## Code availability

The pipeline used for metagenomic quality control, reference-based microbial profiling and gene-centric analyses is available via GitHub at https://github.com/schirmer-lab/metagear-pipeline.
