# Supplementary material for: Microbial collagenase activity is linked to oral–gut translocation in advanced chronic liver disease
Source: Nat Microbiol. 2025 Dec 29;11(1):211–27. doi: 10.1038/s41564-025-02223-0 (PMC12768972; doi:10.1038/s41564-025-02223-0)
Supplement: Supplementary file 1 — The original gel image for Extended Data Fig. 5e. [file 41564_2025_2223_MOESM1_ESM.pdf]

nature microbiology

Supplementary information https://doi.org/10.1038/s41564-025-02223-0

Microbial collagenase activity is linked to oral–gut translocation in advanced chronic liver disease

In the format provided by the authors and unedited

The original gel image for Extended Data Fig 5e

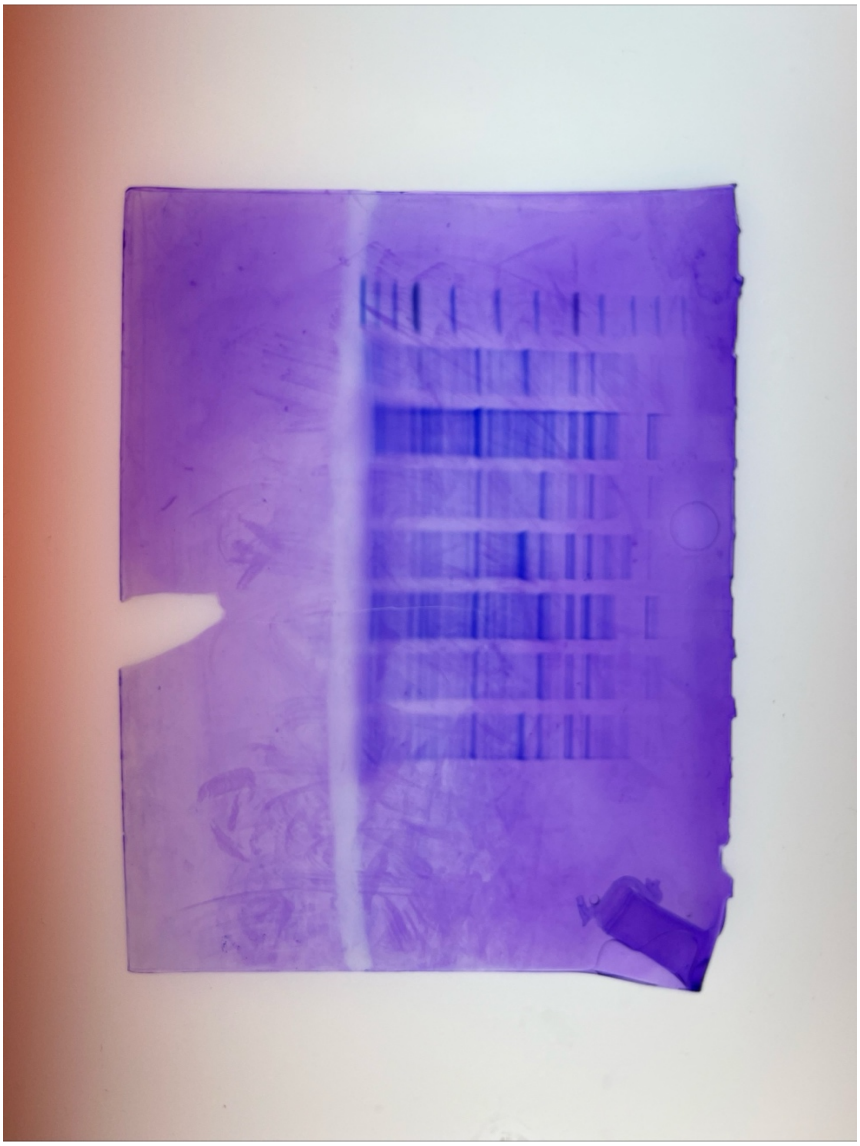

Supplementary Tables:

Table S1: Sample overview, bacterial strains used in this study, and oral-gut translocation events details.
Table S2: Metadata, clinical measurements, microbial profiles and results for *prtC*-based prediction
Table S3: Measurements from CCl_4 mouse experiments
