# Supplementary material for: Microbial collagenase activity is linked to oral–gut translocation in advanced chronic liver disease
Source: Nat Microbiol. 2025 Dec 29;11(1):211–27. doi: 10.1038/s41564-025-02223-0 (PMC12768972; doi:10.1038/s41564-025-02223-0)
Supplement: Supplementary file 2 — Reporting Summary [file 41564_2025_2223_MOESM2_ESM.pdf]

nature portfolio

Corresponding author(s): Melanie Schirmer, Vishal C Patel

Last updated by author(s): Nov 5, 2025

# Reporting Summary

Nature Portfolio wishes to improve the reproducibility of the work that we publish. This form provides structure for consistency and transparency in reporting. For further information on Nature Portfolio policies, see our Editorial Policies and the Editorial Policy Checklist.

## Statistics

For all statistical analyses, confirm that the following items are present in the figure legend, table legend, main text, or Methods section.

| n/a | Confirmed | |
|---|---|---|
| ☐ | ☒ | The exact sample size ($n$) for each experimental group/condition, given as a discrete number and unit of measurement |
| ☐ | ☒ | A statement on whether measurements were taken from distinct samples or whether the same sample was measured repeatedly |
| ☐ | ☒ | The statistical test(s) used AND whether they are one- or two-sided<br>*Only common tests should be described solely by name; describe more complex techniques in the Methods section.* |
| ☐ | ☒ | A description of all covariates tested |
| ☐ | ☒ | A description of any assumptions or corrections, such as tests of normality and adjustment for multiple comparisons |
| ☐ | ☒ | A full description of the statistical parameters including central tendency (e.g. means) or other basic estimates (e.g. regression coefficient) AND variation (e.g. standard deviation) or associated estimates of uncertainty (e.g. confidence intervals) |
| ☐ | ☒ | For null hypothesis testing, the test statistic (e.g. $F$, $t$, $r$) with confidence intervals, effect sizes, degrees of freedom and $P$ value noted<br>*Give P values as exact values whenever suitable.* |
| ☒ | ☐ | For Bayesian analysis, information on the choice of priors and Markov chain Monte Carlo settings |
| ☒ | ☐ | For hierarchical and complex designs, identification of the appropriate level for tests and full reporting of outcomes |
| ☐ | ☒ | Estimates of effect sizes (e.g. Cohen's $d$, Pearson's $r$), indicating how they were calculated |

*Our web collection on statistics for biologists contains articles on many of the points above.*

## Software and code

Policy information about availability of computer code

| | |
|---|---|
| Data collection | No software was used for data collection |
| Data analysis | All tools used for our analyses are publicly available. Further details are provided in the methods section. |

For manuscripts utilizing custom algorithms or software that are central to the research but not yet described in published literature, software must be made available to editors and reviewers. We strongly encourage code deposition in a community repository (e.g. GitHub). See the Nature Portfolio guidelines for submitting code & software for further information.

## Data

Policy information about availability of data

All manuscripts must include a data availability statement. This statement should provide the following information, where applicable:

- Accession codes, unique identifiers, or web links for publicly available datasets
- A description of any restrictions on data availability
- For clinical datasets or third party data, please ensure that the statement adheres to our policy

Metagenomic reads available through SRA (Project ID: PRJEB52891 and PRJNA1307628). All additional data related to this work is also included in Table S2, including clinical metadata and FABP2 measurements, disease scores and MetaPhlAn3 taxonomic profiles

## Research involving human participants, their data, or biological material

Policy information about studies with human participants or human data. See also policy information about sex, gender (identity/presentation), and sexual orientation and race, ethnicity and racism.

| | |
|---|---|
| Reporting on sex and gender | The information regarding the sex and gender were included in table S2 and summarized in table 1 |
| Reporting on race, ethnicity, or other socially relevant groupings | Participants were recruited based on study inclusion criteria. Demographic information was collected, and specific to ethnicity, this was following self-reporting and classified into pre-specified categories that were determined by the study team. Practical measures were taken in line with the principals of good clinical practice to encourage those from ethnic minority groups to participate in the study, including reducing barriers to access the study such as those based around language and level of education. The study cohort represents an inner city urban and suburban population that was predominantly British Caucasian with some ethnic minority representation, with random recruitment based on patients presenting to the study site and meeting study eligibility criteria. |
| Population characteristics | The study cohort was based around liver cirrhosis severity and stage, classified into stable cirrhosis, acutely decompensated cirrhosis, and acute-on-chronic liver failure, with two control cohorts of healthy participants and individuals diagnosed with sepsis and without underlying chronic liver disease. The age and gender of the participants within these five study groups is available, as is data on standard clinical laboratory parameters, physiological measures, pharmacotherapy exposure at the time of biological sampling, and where relevant, disease severity and prognostic composite scores. |
| Recruitment | Patients were consecutively recruited at King's College Hospital after admission to the ward or from the hepatology out-patient clinic. |
| Ethics oversight | The study was granted ethics approval by the national research ethics committee (12/LO/1417) and local research and development department (KCH12-126) and performed conforming to the Declaration of Helsinki. |

Note that full information on the approval of the study protocol must also be provided in the manuscript.

# Field-specific reporting

Please select the one below that is the best fit for your research. If you are not sure, read the appropriate sections before making your selection.

☒ Life sciences ☐ Behavioural & social sciences ☐ Ecological, evolutionary & environmental sciences

For a reference copy of the document with all sections, see nature.com/documents/nr-reporting-summary-flat.pdf

# Life sciences study design

All studies must disclose on these points even when the disclosure is negative.

| | |
|---|---|
| Sample size | Not applicable – non-interventional, cross-sectional, prospective cohort study |
| Data exclusions | Where faecal or saliva samples failed quality control measures for DNA quantity or quality thresholds, these samples were excluded from sequencing and data was not generated for these samples |
| Replication | We have replicated these signals in stool samples from five external validation cohorts (3697 metagenomic data sets in total). |
| Randomization | Not applicable – non-interventional, cross-sectional, prospective cohort study |
| Blinding | Not applicable |

# Reporting for specific materials, systems and methods

We require information from authors about some types of materials, experimental systems and methods used in many studies. Here, indicate whether each material, system or method listed is relevant to your study. If you are not sure if a list item applies to your research, read the appropriate section before selecting a response.

## Materials & experimental systems

| n/a | Involved in the study |
|---|---|
| ☒ | ☐ Antibodies |
| ☒ | ☐ Eukaryotic cell lines |
| ☒ | ☐ Palaeontology and archaeology |
| ☐ | ☒ Animals and other organisms |
| ☐ | ☒ Clinical data |
| ☒ | ☐ Dual use research of concern |
| ☒ | ☐ Plants |

## Methods

| n/a | Involved in the study |
|---|---|
| ☒ | ☐ ChIP-seq |
| ☒ | ☐ Flow cytometry |
| ☒ | ☐ MRI-based neuroimaging |

# Animals and other research organisms

Policy information about studies involving animals; ARRIVE guidelines recommended for reporting animal research, and Sex and Gender in Research

| | |
|---|---|
| Laboratory animals | 10-12-week-old male C57/Bl6 mice |
| Wild animals | Not applicable |
| Reporting on sex | Only male mice were used in our experiments |
| Field-collected samples | Not applicable |
| Ethics oversight | Experiments were approved by the Animal Welfare and Ethical Review Body (AWERB, University of East Anglia, Norwich, UK) and the UK Home Office (Project licence to Beraza: PP9417531, protocol 6). All procedures were carried out following the guidelines of the National Academy of Sciences (National Institutes of Health, publication 86-23, revised 1985) and were performed within the provisions of the Animals (Scientific Procedures) Act 1986 (ASPA). |

Note that full information on the approval of the study protocol must also be provided in the manuscript.

# Clinical data

Policy information about clinical studies

All manuscripts should comply with the ICMJE guidelines for publication of clinical research and a completed CONSORT checklist must be included with all submissions.

| | |
|---|---|
| Clinical trial registration | Not applicable – non-interventional, cross-sectional, prospective cohort study |
| Study protocol | 12/LO/1417/AM01 The Gut-Liver Axis in Liver Disease & Liver Transplantation approved 01-03-2017 by London - Westminster Research Ethics Committee Health Research Authority Research Ethics Service. Not publicly accessible online but available on request. |
| Data collection | Single site study at King's College Hospital Foundation NHS Trust – patients recruited, samples obtained, and data collected between March 2017- August 2019 |
| Outcomes | Primary – saliva and faecal microbiome alpha diversity<br>Secondary – other clinical and lab-based measurements as described in the paper (e.g. plasma FABP2, MELD, Child-Pugh) |

# Plants

| | |
|---|---|
| Seed stocks | *Report on the source of all seed stocks or other plant material used. If applicable, state the seed stock centre and catalogue number. If plant specimens were collected from the field, describe the collection location, date and sampling procedures.* |
| Novel plant genotypes | *Describe the methods by which all novel plant genotypes were produced. This includes those generated by transgenic approaches, gene editing, chemical/radiation-based mutagenesis and hybridization. For transgenic lines, describe the transformation method, the number of independent lines analyzed and the generation upon which experiments were performed. For gene-edited lines, describe the editor used, the endogenous sequence targeted for editing, the targeting guide RNA sequence (if applicable) and how the editor was applied.* |
| Authentication | *Describe any authentication procedures for each seed stock used or novel genotype generated. Describe any experiments used to assess the effect of a mutation and, where applicable, how potential secondary effects (e.g. second site T-DNA insertions, mosiacism, off-target gene editing) were examined.* |
